# Extensive evolution and T cell escape by SARS-CoV-2 in a 2.5-year persistent infection of an immunocompromised host

**DOI:** 10.1016/j.isci.2026.114917

**Published:** 2026-02-05

**Authors:** José Afonso Guerra-Assunção, Ruairi McErlean, Katie Townsend, Selin Cankat, Leonhard M. Flaxl, Shengwei Jamie Tian, Thomas R. Turner, Neema P. Mayor, Judith Breuer, Leo Swadling, David M. Lowe

**Affiliations:** 1 HerpeslabNL, department of Viroscience, Erasmus MC, Rotterdam, the Netherlands; 2 Infection, Immunity and Inflammation, Institute of Child Health, University College London, London, UK; 3 Division of Infection and Immunity, Institute of Immunity and Transplantation, University College London, London, UK; 4 Great Ormond Street Hospital for Children, London, UK; 5 Anthony Nolan Research Institute, Royal Free Hospital, London, UK; 6 UCL Cancer Institute, Royal Free Campus, London, UK

**Keywords:** immunology, immunity, virology, sequence analysis

## Abstract

Prolonged virus-host interaction and suboptimal immunity during persistent SARS-CoV-2 infection of immunocompromised patients enables viral adaptation. We investigated viral evolution and immune escape during a 2.5-year persistent infection in a patient with multiple myeloma and rheumatoid arthritis receiving anti-CD20 therapy. Virus isolated 899-days post-infection revealed an ancestral B.31 lineage with extensive evolution (56 non-synonymous mutations across 20 viral proteins). Many mutations were private or convergent with those seen in other persistent infections and later variants. SARS-CoV-2-specific antibodies were undetectable. Despite prolonged antigen exposure, T cell memory was functional high-in-magnitude and breadth, but with inhibitory receptor expression and dominant spike-specific CD8 response. 38/56 mutations occurred in T cell epitopes, reducing MHC binding or immunogenicity for 69% of CD8 epitopes affected. Importantly, functional assays confirmed T cell escape at 50% (1/2) and 86% (6/7) of CD8 and CD4 epitopes tested *in vitro*. These findings reveal extensive viral adaptation and T cell immune evasion during persistent infection.

## Highlights

• SARS-CoV-2 persisted for 2.5 years in an immunocompromised host, evolving extensively
• Sequencing revealed ancestral B.31 lineage with 56 NS *convergent and unique changes*
• High magnitude functional T cell memory induced, with high inhibitory R expression
• Mutations negatively impacted CD4 and CD8 epitopes, leading to immune escape

## Graphical abstract

**Figure:**
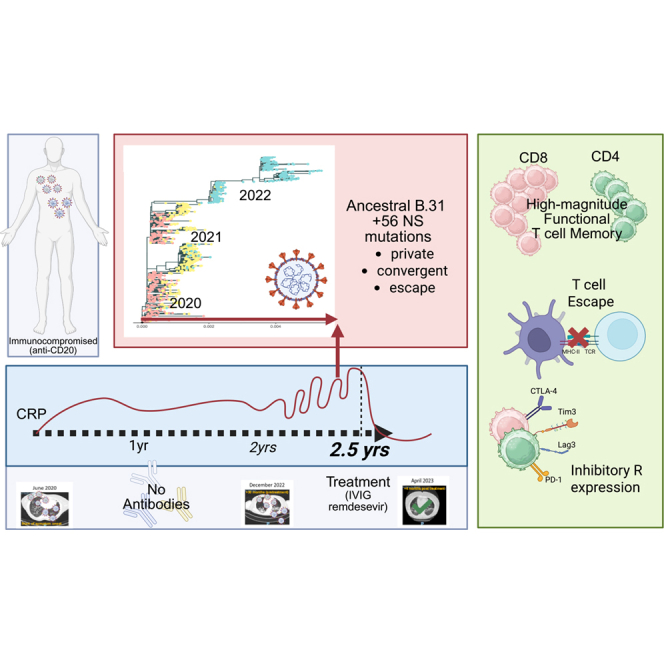

## Introduction

Acute SARS-CoV-2 infections typically resolve within 2 weeks, as the host immune system mounts effective innate and adaptive responses to clear the virus.^1^ However, in immunocompromised patients, who fail to generate robust immune responses due to underlying primary immunodeficiency disorders, immunosuppressive medications, hematologic malignancies, or other conditions, SARS-CoV-2 can persist for months or even years, leading to recurrent symptoms, progressive respiratory decline, and an increased risk of mortality.^2^

Prolonged infection allows ongoing viral replication under weak immune selection pressure in the host, during which time mutations can occur.^3^^,^^4^^,^^5^^,^^6^^,^^7^ Many mutations are expected to be detrimental to the virus, but some can provide fitness advantages and may be selected for. This led to the hypothesis that prolonged infections in immunocompromised individuals may give rise to novel variants of concern, and that host-adapted and immune-evading variants can then be transmitted back to the population through prolonged shedding. Indeed, there is significant interest into whether the Omicron variant may have evolved during persistent infection in an immunocompromised host.^8^^,^^9^

Characterizing viral evolution and immunity during prolonged infections is crucial to understanding viral adaptation, immune evasion mechanisms (including T cell exhaustion and escape), the pathophysiology of COVID-19 and potentially mechanisms underlying Long-COVID.^3^^,^^10^^,^^11^

In a previous study, we characterized a set of clinical outcomes for immunocompromised patients receiving intravenous immunoglobulin (IVIG) treatment.^12^ Here, we describe the extensive intra-host evolution of an isolate related to an ancestral B.31 lineage during a 2.5-year infection. We demonstrate that strong, high quality T cell memory is generated during persistent infection, dominated by spike-specific CD8 T cell responses. We do not identify mutations that would be expected to lead to resistance to monoclonal antibodies or antivirals, but we observe loss of recognition by host T cells of the emerging virus at several epitopes, demonstrating T cell escape.

## Results

### Clinical characterization of a 2.5-year infection

**Figure 1:**
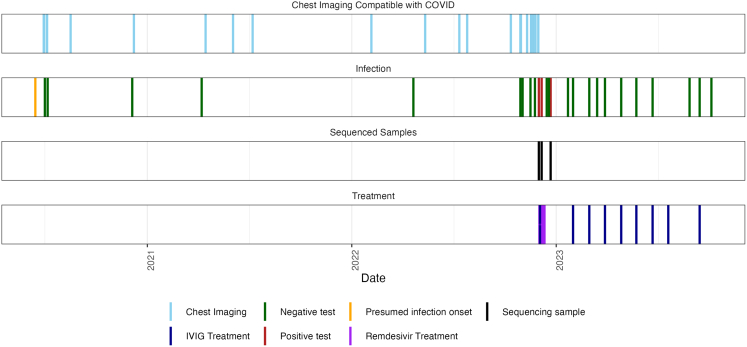
Timeline of clinically relevant events during persistent SARS-CoV-2 infection Vertical lines indicate key clinical events over the 2.5-year infection period. The presumed infection onset is marked at the beginning of the timeline. Chest imaging: CT scans and chest X-rays demonstrating typical changes consistent with COVID-19 (June 15, 2020 putative infection onset and time of first A&E admission; see also Figures S1 and S2). Infection status: Negative (green) and positive (red) PCR tests on nasal swabs, sputum samples (positive on December 1, 2022, December 6, 2022, and December 22, 2022), and LFTs. Treatment: Administration of IVIG (purple) and remdesivir (10-day course, magenta; December 6, 2022). Sequencing samples (December 1, 2022): Specimens collected for SARS-CoV-2 viral sequencing (Table S2). Note persistent radiological changes despite intermittent negative PCR results throughout the infection period. Symptom resolution, normalization of CRP, and seroconversion occurred mid-January 2022.

A patient with a history of multiple myeloma and rheumatoid arthritis, treated with prednisolone (corticosteroid) and rituximab (anti-CD20 monoclonal antibody, mAb), suffered recurrent relapsing-remitting episodes of cough, breathlessness, and fever from July 2020. They required repeated hospital reviews and admissions over the ensuing 2.5 years with multiple investigative workups for sepsis and fever of unknown origin. Infective screens were repeatedly negative including sputum for microscopy, culture, and sensitivity (MCS), prolonged sputum cultures for acid fast bacilli and tuberculosis polymerase chain reaction (PCR), bronchoalveolar lavage gram stain and sputum culture, aerobic and anaerobic blood cultures, respiratory viral swabs (for seasonal coronaviruses, respiratory syncytial virus, influenza A and B, parainfluenza 1–4, parechovirus, respiratory adenovirus, rhinovirus, human metapneumovirus, and respiratory enterovirus RNA), atypical pneumonia screening, and fungal and viral blood markers (Table S1). SARS-CoV-2 PCR, including on bronchoscopy, was also consistently negative (Figure 1). Blood tests revealed persistently raised inflammatory marker C-reactive protein (CRP; 57 successive measurements between June 29, 2020 and December 9, 2022 above upper-limit of normality; Figure S1); procalcitonin when measured was <2 ng/mL, suggesting low likelihood of a bacterial cause. Lung imaging demonstrated multifocal ground glass changes in a changing pattern, typical of severe SARS-CoV-2 infection (Figures 1 and S2).

They were admitted to hospital in December 2022 due to acute respiratory deterioration and required intensive care admission. At this time, SARS-CoV-2 was identified on nasopharyngeal swab and successfully sequenced. The patient was receiving anti-CD20 medication and did not have detectable anti-spike antibody, despite multiple vaccinations (all given after development of first symptoms and suspected time of infection) and prolonged infection. They were treated with intravenous remdesivir (200 mg on day 1 then 100 mg daily for a total of 10 days) and IVIG (1 g/kg over 2 days) with clinical recovery, detection of (passively acquired) spike antibody in serum, conversion of SARS-CoV-2 PCR tests to negative and consistent normalization of CRP levels for the first time since the episode began in mid-2020 (Figure S1). Overall, the clinical presentation and extensive follow-up are highly suggestive of a persistent viral infection.

They remain on prednisolone (maintenance dose 10–15 mg daily) and have received further rituximab infusions 6-monthly for their rheumatoid arthritis. Given the above presentation they were worked up for secondary immunodeficiency and have been maintained on replacement immunoglobulin therapy indefinitely. However, they had ongoing breathlessness, wheezing, and hypoxia, with persisting lung damage on imaging consistent with chronic airways disease (Figure S2). This has been associated with recurrent superimposed bacterial infection more recently, with isolation of bacterial pathogens in sputum (*Haemophilus influenzae* and *Pseudomonas aeruginosa*) and frequent need for antibiotic therapy. Notably, prior to the SARS-CoV-2 infection they had no clinical or radiological lung disease.

### Virus isolated during persistent SARS-CoV-2 infection accumulates convergent and infection specific mutations

Despite a clinical presentation indicative of SARS-CoV-2, virus was repeatedly not detected by lateral flow test (LFT) or PCR on nasal swabs, sputum, or even bronchoscopy, as has been described for other persistent infections. Virus was isolated from this patient by nasal swab 899 days after suspected onset of SARS-CoV-2 infection (PCR cycle threshold 32; Zenodo: https://zenodo.org/records/17341064 and GISAID, hCoV-19/England/22K457422/2022). Sequencing quality control failed when viral loads were lower, with 1/3 samples from this patient passing QC filters for downstream analysis (Table S2). UShER (Ultrafast Sample placement on Existing tRees^13^) was used to determine the rapid phylogenetic placement of the genomic sample within an existing reference 2 million SARS-CoV-2 phylogenetic tree using publicly available sequence data from GISAID (Global Initiative on Sharing All Influenza Data^14^). In agreement with the first onset of respiratory symptoms observed in the patient in mid-2020, the genome sequence was assigned as “Pango” lineage B.31 (Emerging clade: B (19A), reassigned from B.2.5. Circulating in Africa, Asia, Oceania, North/South America and Europe [UK] from March 2020 to July 2020). UShER placement revealed the closest ancestral sequence as a virus isolate sampled from Wales in April 2020 (Wales/PHWC-28F23/2020|2020-04-02, EPI_ISL_445459). Only 259 virus genome records corresponding to the B.31 Pango lineage were available in the GISAID database, predominantly sampled from the UK, and it was last sequenced in May 2020 (database queried October 2025^15^). In summary, the viral sequence identified at day 899 of suspected infection had the sequence characteristics of a variant circulating at the time of clinical presentation and suspected infection, which had not been sequenced elsewhere globally for over 2 years.

**Figure 2:**
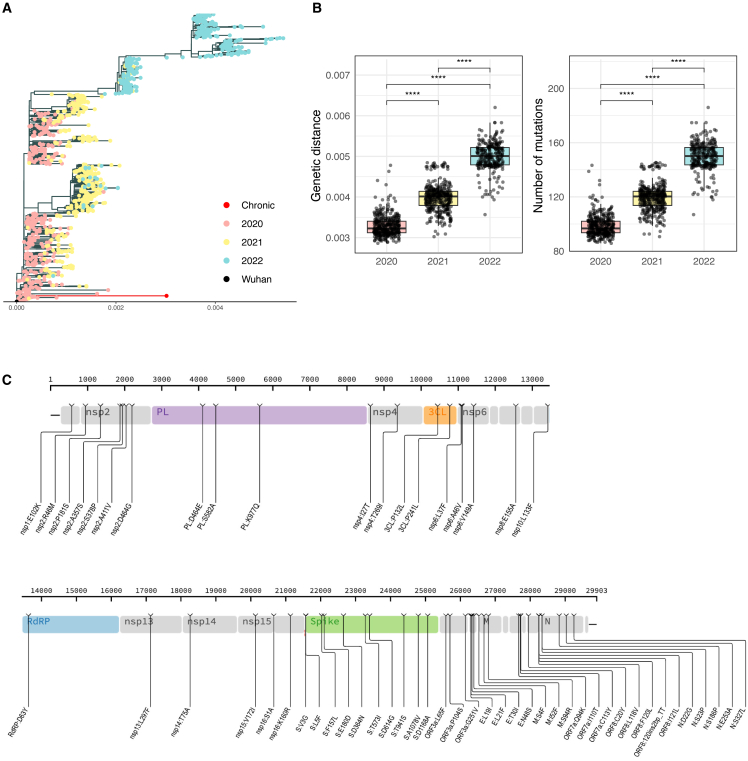
Phylogenetic placement and mutational landscape of the SARS-CoV-2 sequence detected at day 899 of infection (A) Maximum-likelihood phylogenetic tree of 997 global SARS-CoV-2 genomes (2020–2022) rooted on Wuhan-Hu-1 (black), showing the chronic sequence (red) branching among early 2020 lineages. Tip colors represent year of collection (2020 = pink, 2021 = yellow, 2022 = blue). (B) Genetic distance (proportion differences) and number of mutations (absolute differences) of the chronic sequence relative to SARS-CoV-2 genomes from 2020, 2021, and 2022 (Wilcoxon rank-sum test, all ∗∗∗∗*p* < 2×10^−16^), indicating progressive divergence from early lineages. Bars, median plus IQR, and Tukey whiskers at 1.5xIQR. (C) Distribution of non-synonymous mutations in the day 899 sequence seen across the SARS-CoV-2 viral proteome at consensus level relative to closest public sequence. Amino acid position is shown above. Mutations listed below as affected protein, ancestral amino acid, position in protein, and variant amino acid. 3CL, 3C-like protease; E, envelope; M, membrane protein; N, Nucleoprotein; NSP5; NSP, non-structural protein; ORF, open reading frame; PL, papain-like protease NSP3; RdRP, RNA-dependent RNA-polymerase.

To investigate the similarity between the virus isolated during the chronic infection and sequences from early SARS-CoV-2 lineages, a maximum-likelihood tree rooted on Wuhan-Hu-1 was constructed and color-coded by year (Figure 2A). The chronic sequence branched among early 2020 lineages, consistent with an origin linked to early pandemic viruses. The 2021 sequences were broadly distributed between 2020 and 2022 clades, reflecting transitional variants spanning these periods, while the 2022 genomes formed distinct, more derived clusters consistent with the diversification of later variants. To quantify these relationships, pairwise genetic distances and mutation counts were compared between the chronic sequence and genomes from each year (Figure 2B). Both metrics showed a clear, stepwise increase from 2020 to 2022, indicating progressive divergence over time (Wilcoxon rank-sum test, all *p* < 2×10^−16^). These findings demonstrate that the viral sequence identified is most closely related to early 2020 lineages and increasingly distant from later variants, consistent with its phylogenetic placement.

To determine the number of mutations accumulated in the genome, a map of single nucleotide polymorphisms (SNP) relative to the closest matching reference sequencing was created based on a pairwise comparison of the genetic identity between consensus genomes (Figure S3). In contrast to other shorter persistent infections we studied,^12^ where the number of accumulated mutations observed relative to the closest related sequence were fewer than 15, this sample differed from its closest public B.31 relative by 79 mutations. Out of these, 56 were non-synonymous (Figure 2C). The virus had 5 mutations characteristic of ancestral viruses: ORF1ab:L3606F, ORF1ab:D54Y, ORF3a:G25IV, ORF8:S84L, and N:D22G (Figure 2C).

Despite intra-host evolution, the treatment regimen administered (remdesivir plus IVIG) was sufficient to finally control the infection, as assessed by sustained viral clearance post-treatment (Figure 1). This patient was, however, subsequently reinfected with a XEC.2 strain of SARS-CoV-2 in October 2024 (ENA Biosample: SAMEA117560877), demonstrating that the immunity developed during the persistent infection did not protect upon subsequent viral challenge with a different variant. However, this new infection event was brief with rapid resolution of clinical symptoms and PCR positivity. The virus isolated from this subsequent infection did not share any mutations or regions of similarity suggestive of recombination or further persistence of the virus isolated during primary infection.

### 2.5-Year infection leads to extensive mutations across the viral genome

We first investigated whether the genetic variation seen within the sequence taken at day 899 could have been the result of viral recombination during the persistent infection. At the time of sequencing (December 2022), the dominant variants circulating in the United Kingdom and Europe were BA.5, BQ.1, and XBB. We found no evidence of contiguous mutation stretches or lineage defining mutations shared between the patient sample and globally circulating viruses (Figures 2 and S3). This finding militates against viral recombination with newer reinfecting variants as the origin of the polymorphisms observed, instead strongly suggesting that viral genetic variation within this individual evolved during chronic infection.

Next, we considered whether the mutations arising in our patient were common to persistent infections, indicative of convergent evolution. Two studies have compiled data from several sources on the mutations acquired during persistent infections that may indicate evasion of adaptive immunity or adaptation of the virus in the specific setting of chronic infection (where selection pressures differ to acute resolving infections).^10^^,^^16^ Of the 21 mutations that have been observed in multiple persistent infections (168 genomes, as compiled in Wilkinson et al.^16^) two emerged in the patient described here: E:T30I and NSP3:K977Q (also observed as a lineage defining mutation for the P.1 gamma variant of concenr [VOC]). Wilkinson et al. described the E:T30I mutation as a “sensitive marker” of persistent infection due to its repeated appearance in different studies and no association with a particular source, geographical region, or SARS-CoV-2 lineage.^16^ Mutations within the spike protein, V3G and A1078V seen in our patient have also been reported previously in persistent infection.^10^^,^^11^ The most comprehensive study of persistent infection using repeated PCR positivity also identified ORF8 mutation I121L as being recurrently identified in prolonged infections.^17^ Overall, a subset of the amino acid changes seen in the persistent infection described here have been previously described in a small number of other persistent infections, suggesting convergent evolution that might confer a fitness advantage in this setting.

Some of the non-synonymous mutations acquired during this persistent infection are rarely observed elsewhere globally (16 mutations observed in <1,000 sequences sampled globally since the beginning of the pandemic to May 2025); however, several are characteristic of VOCs or sub-lineages that subsequently emerged, such as: S:D614G, NSP3:K977Q, NSP6:V149A, S:F157L, S:T573I, ORF3a:P104S, and NP:S327L. In particular, Spike D614G mutation has been widely studied, marks the B.1-like genotypes^18^ and was present in almost all sequences post-May 2020, but was lacking in B.31. This suggests that features of emerging variants in the general population may be predicted from studies of intra-host evolution in chronically infected individuals.^19^

The spike protein is the main target of humoral immunity, in particular for neutralizing antibodies, but is also immunodominant within T cell responses seen after infection.^20^ This protein also plays a key role in viral entry to the host cell, and it is therefore the main viral protein undergoing positive selection. Analysis of the frequencies of the spike mutations acquired in our patient within the GISAID database revealed that most of the nine fixed NS spike mutations were not widely detected in general circulation (mutations E180D, D364N, A1078V, and D1168A were observed in approximately 62, 3, 125, and 7 sequences per million, respectively), suggesting they may reduce viral fitness.^21^ The spike mutations V3G, S:F157L, T573I, and D614G, conversely have circulated more widely, however, in no other publicly available sequence have these 4 mutations been observed together.

In this seronegative patient, mutations in structural proteins were not associated with antibody escape, and mutations were not enriched in the receptor binding domain, the main target of neutralizing antibodies. Mutations did not appear to be randomly distributed across viral proteins, however, as they were less common than expected in the non-structural proteins (NSPs) of ORF1ab, which accounts for ∼71% of the viral genome but contained just 45% of the mutations. This observation is in line with the notion that non-synonymous mutations are less tolerated on NSPs, as these are likely purged through purifying selection, due to functional and structural constraints in proteins that play a vital role in viral replication.^22^^,^^23^

As described above, no protease or polymerase inhibitors or SARS-CoV-2-specific mAb were administered prior to the virus being isolated at day 899 of infection. As expected, none of the 9 mutations in spike observed during persistent infection have been described as mAb resistance mutations (Stanford CoV-RDB^24^). Only mutation D364N falls within the receptor binding domain (RBD) but it does not directly disrupt known RBD antibody binding sites, and as such is not predicted to cause antibody escape.^25^ The mutations that fall in NSP5 (3CLpro) and NSP12 (RdRp) have not been linked to resistance to 3CLpro or polymerase inhibitors.^24^

These data suggest that a B.31 virus (early Wuhan-hu-1-like isolate) has undergone extensive evolution during an infection that has lasted over 2.5-year, leading to recurrent emergence of mutations, some of which have been seen previously in persistent infections, others seen in subsequently emerging VOCs, but also a large set of unique (private) mutations.

### Robust and functional T cell memory is established despite persistent infection

Chronic viral infections and associated persistent antigen exposure can drive T cells to become dysfunctional and “exhausted.”^26^ SARS-CoV-2 is not adapted to persist in immunocompetent individuals, but whether persistence in immunocompromised patients leads to virus-specific T cell exhaustion has not been well studied.

**Figure 3:**
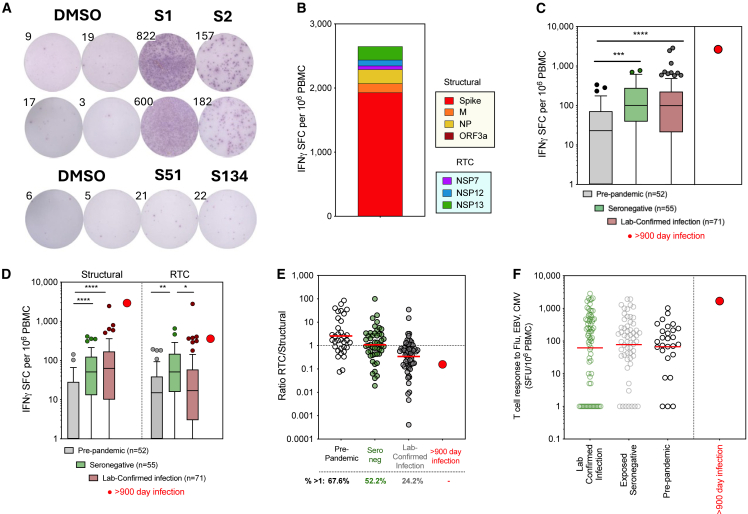
Magnitude and specificity of T cell memory responses generated by 899-day infection (A) *Ex vivo* IFNγ-ELISpot well images. DMSO control unstimulated wells and wells stimulated with overlapping peptides covering S1 and S2 region of Spike (duplicates shown), or single peptides S51 and S134. (B) Total magnitude of SARS-CoV-2-specific memory T cell response to structural proteins Spike, membrane (M), nucleoprotein (NP) and open reading frame 3a (ORF3a) and RTC proteins (NSP7, NSP12 polymerase, and NSP13 helicase) colored by protein targeted and measured after persistent infection (>900 days). (C and D) Total magnitude of SARS-CoV-2-specific T cell response (C) or T cell response to Structural and RTC proteins (D) in pre-pandemic samples (pre-August 2019), in exposed healthcare workers (HCW) who remained seronegative, including abortive infections, and in HCW with laboratory confirmed SARS-CoV-2 infections (samples 4 months post-exposure/infection in June to July 2020) for comparison to T cell response in persistently infected patient. (E) Ratio of the magnitude of the T cell response to RTC/structural T cells. Percentage of cohort with a response above 1 (stronger response to RTC than structural proteins) shown below. (F) Magnitude of T cell response to a pool of epitopes from Flu, EBV, and CMV. (B–E) Subset of data previously published in Swadling et al.^22^ (A–F) IFNγ-ELISpot. (C and D) Box and Whisker, Tukey. (C–F) Statistical analysis was performed using Kruskal-Wallis tests with Dunn’s correction. ∗*p* ≤ 0.05; ∗∗*p* ≤ 0.01; ∗∗∗*p* ≤ 0.001; ∗∗∗∗*p* < 0.0001. (E and F) Bars, geomean.

We next assessed what T cell immunity was generated by this long-term persistent infection without seroconversion. The memory T cell response targeted all SARS-CoV-2 proteins tested but was strongest to the spike protein (Figures 3A and 3B). When quantifying the SARS-CoV-2-specific T cell response in the blood, the total magnitude was larger than that detected in pre-pandemic samples, in exposed-seronegative healthcare workers,^22^ and larger than all but one response seen 4 months after infection with the emergent Wuhan-hu-1 sequence (Figure 3C, *n* = 71), despite it being measured here ∼1 year after resolution of infection and at the time of corticosteroid use. This high magnitude T cell response was dominated by structural protein-specific T cells, as has been described for memory responses after infection with Wuhan-hu-1 SARS-CoV-2^27^^,^^28^ (Figure 3D); however, a T cell response to the NSPs that make up the core of the viral replication-transcription complex (RTC) was also seen. RTC-specific T cells have been associated with early control of viral replication, being enriched before and after abortive SARS-CoV-2 infection both in natural history studies and human challenge.^22^^,^^29^ The ratio of RTC- to structural protein-specific T cells was lower during persistent infection than seen after an acute-resolving Wuhan hu-1 infection, suggesting prolonged infection biases the T cell response toward spike and structural proteins (Figure 3E). This patient had a high frequency of total CD8 T cells (66.0% of CD3, versus CD4 29.4% of CD3) and a low frequency of naive T cells (Figures S4A–S4C). The T cell response to control antigens from influenza (Flu), EBV, and CMV was also relatively high in magnitude, in the 97th percentile of all healthy donors tested, which may be due to a higher frequency of T cells in the blood relative to other PBMC subsets for this immunocompromised patient (1,678 SFU/10^6^ PBMC; Figures 3F and S4A).

**Figure 4:**
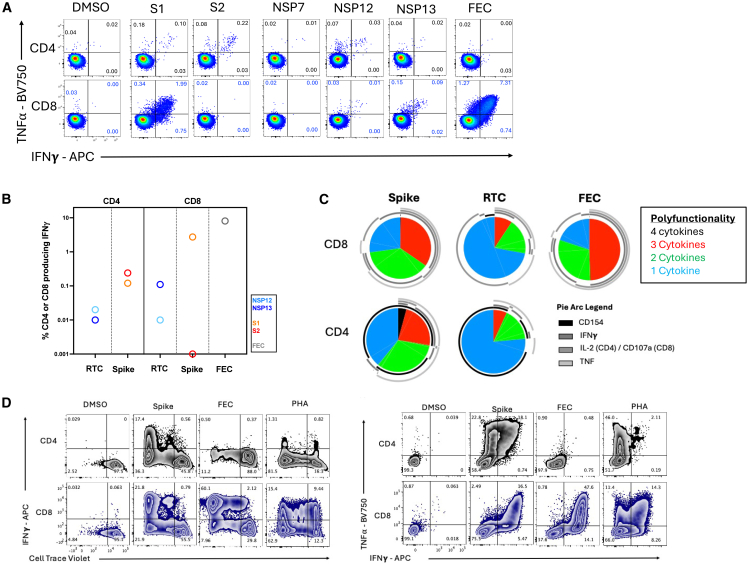
*Ex vivo* functionality and proliferative potential of T cell memory generated by a 899-day persistent infection (A) The IFNγ and/or TNF⍺ producing CD4 and CD8 T cell memory response to SARS-CoV-2 proteins after persistent infection. Percentage of CD4 or CD8 is shown. Spike divided into S1 and S2 peptide pools. DMSO, unstimulated control. FEC, response to Flu, EBV, and CMV epitope pool. (B) Summed CD4 or CD8 IFNγ+ response to spike (S1, S2) or the replication-transcription proteins (RTC; NSP7/12/13). (C) The proportion of SARS-CoV-2-specific CD4 or CD8 T cells to spike or RTC that co-produce several cytokines/effector molecules are shown as pie charts. Pie arcs show the proportion producing each single cytokine. (D) Proliferation and expansion of SARS-CoV-2-specific memory CD4 and CD8 T cells post-persistent infection when *in vitro* stimulated with Spike, Flu EBV and CMV epitopes, or PHA, as shown by CTV dilution and IFNγ production after 8-day stimulation. Percentage of CD4 or CD8 shown for each quadrant. PHA, phytohemagglutinin.

We next used surface marker and intracellular cytokine staining (ICS) to determine whether these SARS-CoV-2-specific T cells were CD4 or CD8, and to confirm their functional capacity. ICS confirmed the immunodominance of spike relative to NSPs (Figure 4A; Gating strategy; Figure S4A). Spike-specific T cells were predominantly CD8 (∼2.73% of CD8 and ∼0.36% of CD4 T cells IFNγ+) and targeted the S1 region. CD4 T cells to both S1 and S2 regions were also detected. T cell responses to RTC were lower in magnitude but more balanced between CD4 (∼0.06% IFNγ+) and CD8 (∼0.05% IFNγ+) (Figure 4B). SARS-CoV-2-specific CD4 and CD8 memory T cells were highly functional, producing the antiviral and immunomodulatory cytokines IFNγ, TNF, and IL-2, or expressing CD154 upon peptide stimulation *in vitro* (Figure 4C). SARS-CoV-2-specific T cells were almost exclusively effector memory phenotype (Tem; CD45RA-CCR7-; Figure S4D).

We next assessed T cell proliferative capacity. Both CD4 and CD8 SARS-CoV-2-specific T cells generated by persistent infection showed significant expansion *in vitro* in response to spike peptide pool stimulation (∼55-fold for CD4, ∼8.3-fold for CD8 for the IFNγ+TNF+ population comparing *ex vivo* cells to *in vitro* expanded cells), similar to the CD8 T cell response to influenza, EBV and CMV (∼6.5-fold for CD8 IFNγ+TNF+; Figure 4D). Finally, we investigated the activation-induced marker expression and phenotype of spike-specific memory T cells after long-term chronic infection (Gating strategy; Figures S5A and S5B). Bulk CD4 and CD8 T cells in this immunosuppressed individual did not show any defect in response to anti-CD3/CD28 (Figure S5C), and they did not express higher levels of the co-inhibitory markers CTLA-4, Lag-3, Tim-3, PD-1, or master transcription factor TOX than bulk T cells from healthy controls; however, the SARS-CoV-2 spike-specific T cell memory in the blood after chronic infection showed higher expression of all coinhibitory markers, but without expression of one of the master transcription factor of exhaustion, TOX (Figures S5D and S5E).

Overall, this indicates that SARS-CoV-2-specific memory T cells retained their proliferative capacity, polyfunctionality and their ability to respond *in vitro* to peptide stimulation; however, their phenotype was altered, with higher expression of coinhibitory markers, potentially reflecting lower *in vivo* responsiveness.

### Mutations acquired during persistent infection are predicted to impact the presentation and immunogenicity of T cell epitopes

Two major mechanisms by which variable viruses escape T cell recognition are: (1) the introduction of mutations that reduce or abrogate peptide binding to MHC, reducing presentation of viral peptides to T cells (in particular mutations at 1,2 and c-terminal residues); and (2) by introducing mutations that reduce TCR recognition of peptide-MHC complexes. To determine if the mutations acquired here would impact T cell recognition of virally infected cells we first determined if the 56 mutations fall within known T cell epitopes. Known epitopes were defined here as in Grifoni et al.,^20^ as optimal length (8-14mer class I or 12-25mer class II) peptides which elicit a response in functional T cell assays (as opposed to MHC binding only) and for which the CD4/CD8 status of responding cells is known (Tables S3 and S4). A third of the mutations (18/56) had no impact on known epitopes; however, 38 mutations fell within 38 CD4 (Table S3) and 55 CD8 epitopes (Table S4). HLA typing was performed on the patient (Table S5), showing that of the epitopes in which mutations fall 17 are known to be restricted by HLA-alleles carried by the donor, with a further 18 being predicted to be binders to the patient’s HLA (35/55 epitopes in total) for CD8 epitopes. Peptides are often more promiscuous for MHC class II alleles; however, the prediction algorithms are less accurate. Seven of the 38 epitopes affected by mutations seen during this persistent infection are known to be restricted by the patient’s class II HLA alleles, with a further 3 epitopes being predicted to be binders to the patient’s HLA (10/38 in total).

We used machine learning tools to predict the impact of mutations on peptide presentation and immunogenicity. Mutations arising during this long-term persistent infection are predicted to negatively impact peptide binding and presentation to CD8 T cells for 53% (9/17) epitopes known to be restricted by the patient’s HLA alleles (Table S4 and Figure S6).

Prediction of peptide binding to MHC class II is less accurate with current models. Of all the known CD4 epitopes which are affected by mutations seen at day 899 of infection, regardless of HLA restriction, only 13 of the validated SARS-CoV-2 epitopes were predicted to be binders for MHC class II (Table S3); of these, 6 were unaffected by the acquired mutations, 1 was negatively impacted and 5 had improved peptide binding. Only one of the CD4 epitopes known to bind the patient’s HLA alleles was predicted to be a binder by NetMHCIIpan and the variant sequence did not lead to predicted loss of MHC binding.

The mutations acquired during this persistent infection negatively impacted immunogenicity scores for 51% (18/35) of the CD8 T cell epitopes known or predicted to be restricted by this patient’s HLA alleles (Table S6).

Overall, the mutations are expected to negatively impact T cell recognition by reducing either presentation and/or immunogenicity for 69% (24/35) of CD8 epitopes known or predicted to be presented by the patient’s HLA alleles.

### Mutations acquired during persistent infection led to T cell escape

**Figure 5:**
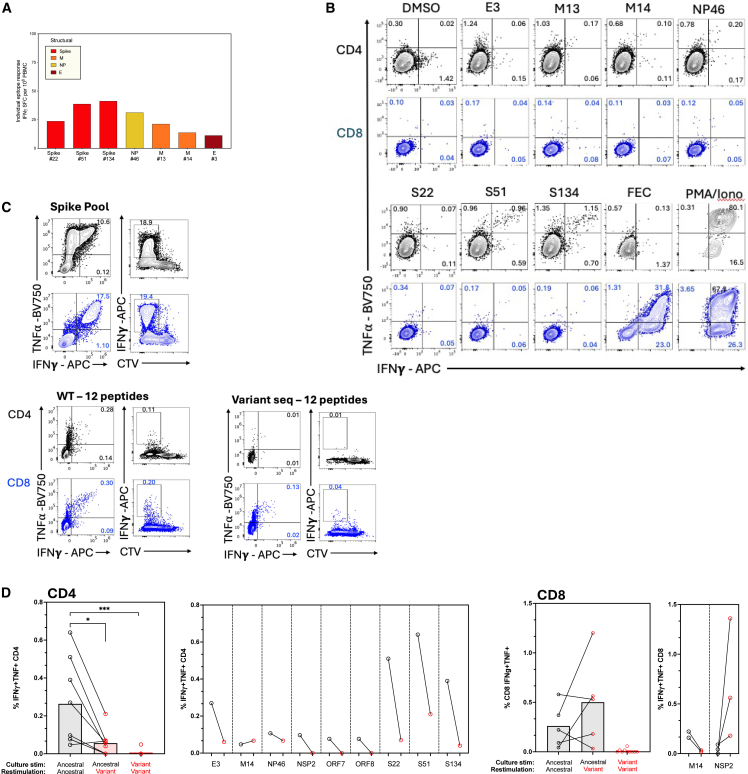
Failure of host T cells to recognize emerging virus (A) *Ex vivo* magnitude of the T cell response to individual ancestral sequence peptides in which mutation arose over persistent infection by IFNγ-ELISpot. (B) Magnitude of the CD4 and CD8 T cell responses after 10-day *in vitro* peptide expansion with individual ancestral sequence peptides. Percentage of CD4 and CD8 producing IFNγ, TNF, or both are shown. (C) Magnitude of IFNγ+, IFNγ+TNF+, and CTV^lo^IFNγ+ CD4 and CD8 T cells after 8-day expansion with a pool of 12 peptides corresponding to ancestral sequence epitopes or variant sequence epitopes containing mutations in the virus isolated at day 899 of infection. (D) Magnitude of the IFNγ+TNF+ CD4 or CD8 T cell response after 10-day *in vitro* peptide stimulation with individual epitopes using ancestral sequence or variant sequence peptides for culture and re-stimulation on day 9. Summary data showing all T cell responses that were detectable (left) or shown for individual peptides (right) for CD4 then CD8 T cells. Duplicate and triplicate stimulations are shown for CD8 T cells for M14 and NSP2, respectively. (D) Bars, mean. Statistical analysis was performed using Kruskal-Wallis tests with Dunn’s correction. ∗*p* ≤ 0.05; ∗∗∗*p* ≤ 0.001. Peptide sequences shown in Table S7.

To determine if the mutations acquired during the persistent infection led to functional loss of recognition and T cell escape, we took a subset of immunodominant epitopes^20^ in which mutations arose and measured whether the patient had a detectable T cell response to these. As expected, T cell responses to individual epitopes were low magnitude *ex vivo* (Figure 5A; Table S7); however, functional T cells could be readily expanded *in vitro* for a subset of these epitopes (Figure 5B). Next, we tested whether the patients T cells could recognize the mutant versions of each epitope that had arisen during persistent infection. We expanded T cells with a pool of 12 peptides of ancestral or variant sequence, showing that the magnitude of the response to the ancestral infecting virus sequence was higher than the response to the variant peptide pool corresponding to the virus sequence at day 899 of persistent infection (Figure 5C).

When considering individual epitope for which a T cell response was detectable, 6/7 of the CD4 and 1/2 CD8 epitopes T cell recognition was almost completely abrogated against the variant peptide (Figure 5D). The mutant version of M14 was, however, equally well recognized by CD4 T cells as the ancestral sequence. A mutation in NSP2 (A411V; TSDLATNNLVVMVY) lead to better CD8 T cell recognition *in vitro* (Figure 5D). Attempted T cell expansion *in vitro* with the mutant version of these epitopes, however, did not lead to T cell expansion except for CD4 T cells recognizing M14 and a very low magnitude CD8 T cells response to NSP2 variant (0.01% variant expansion vs. 0.7% of CD8 T cells on average for expansion with ancestral peptide; Figure 5D), confirming the lack of recognition of most mutant variants and demonstrating that mutations did not introduce *de novo* epitopes. Therefore, mutations arising during a 2.5-year infection led to loss of epitope recognition and T cell escape.

## Discussion

Here, we have identified the longest SARS-CoV-2 infection described to date, persisting over 2.5-year based on clinical symptoms, lung pathology, and viral sequence characterization despite the virus being undetectable in respiratory samples for the majority of the infection.

Persistent SARS-CoV-2 infections that last so long are rare and tend to occur in people with significant immunocompromise, with the most comprehensive study to date identifying just 10 of ∼94,000 infections lasting more than 100 days and none longer than 193 days when relying on PCR positivity to determine duration of infection.^17^ It is important to note that it is uncommon for patients on anti-CD20 therapy to develop persistent infection, with innate immunity and T cells sufficient to mediate control in most cases,^30^^,^^31^ and so additional factors likely contribute to the inability of this patient to naturally resolve SARS-CoV-2 infection, as demonstrated by this rare long-term infection. Due to multiple comorbidities and immunosuppressive treatment, it is unclear which factors contribute most to viral persistence in this case.

It is difficult to identify and study persistent infections that do not result in detectable virus in bronchoalveolar lavage or nasal samples; however, postmortem studies have identified persistence of virus in bronchial glands and chondrocytes, without detection in respiratory epithelium, in individuals who died due to pneumonia.^32^ Viral rebound is common in persistent infections,^17^ and could be the result of acquired adaptation and immune evasion. This may also explain why detectable virus was only seen at day 899 in our chronic infection despite regular testing and constant clinical indicators of infection. This highlights the importance of using a mixture of molecular and clinical tools to identify and study viral persistence, especially given the risk of irreversible lung damage as seen in our patient.

The rate of mutations observed during persistent infections has been estimated to be ∼2× higher than that seen when tracking at the population level.^4^^,^^11^^,^^33^ Transmission is characterized by a narrow bottleneck^34^ and a period of low mutation rate has been observed in “short” persistent infections in immunocompetent patients (56–100 days^17^), suggestive of weak selective pressures. In contrast, persistent infections >100 days, almost invariably in immunocompromised patients, releases the virus from the constraints of transmission bottlenecks and give the opportunity to adapt and acquire immune escape mutations. As the longest SARS-CoV-2 infection described to date, an extensive mutational profile was observed, with 79 mutations, resulting in 56 non-synonymous and one insertion affecting almost all viral proteins. These mutations were indicative of an ancestral strain that had acquired an extensive array of mutations. The virus mapped to the B.31 lineage, which had not been identified since July 2020, and importantly it showed no signs of recombination or characteristic mutations of the variants circulating at the time of isolation (in 2022). The chronic infection viral sequence clustered with 2020 sequences and the genetic distance and number of mutations increased compared to 2021 and 2022 sequences. It carried several key mutations of ancestral stains (ORF1ab:L3606F, ORF1ab:D54Y, ORF3a:G25IV, ORF8:S84L, and N:D22G), a set of convergent mutations that had been previously seen in persistent infections (E:T30I and NSP3:K977Q ORF8 mutation I121L^16^^,^^17^), as well as a set of unique or private mutations. Overall, the virus isolated at the end of this long-term infection had the backbone of viruses circulating in 2020, around the time of clinical presentation, but with a large number of private mutations and mutations associated with persistent SARS-CoV-2 infection.

Persistent infection can offer a window into how SARS-CoV-2 may evolve at an inter-host or population level, with this viral isolate acquiring several mutations that became fixed in later variants (in particular S:D614G and NSP3:K977Q). The now almost universal D614G mutation in spike enhances viral transmission by increasing S protein stability and binding affinity.^18^^,^^35^ The absence of D614G in the closest sequence to this sample suggests that this infection occurred when D614G was not yet ubiquitous, although it is impossible to confirm if it appeared independently in this patient, as the data suggest. As immunocompromised patients have been suggested as the origin of VOCs,^17^^,^^36^ this underpins the importance of monitoring their infections for viral clearance.^37^

An important consideration for viral evolution in antibody-deficient patients is that the lack of humoral immune pressure reduces selective forces shaping the virus. Without an effective antibody response, mutations enabling antibody escape do not provide a replicative advantage. However, some immunocompromised patients retain marginal B cell function sufficient to exert weak antibody pressure^38^ or might receive inadequate titers of antibody via therapeutic products. Here, we saw no mutations that have previously been associated with antibody evasion and no enrichment for mutations in spike and its receptor binding domain, in line with a complete lack of SARS-CoV-2-specific humoral immunity prior to virus isolation. Administration of IVIG here led to control of infection and so did not contribute to selection of antibody evasion mutations. IVIG treatment has shown success in treating prolonged COVID-19 infection in immunocompromised patients.^12^^,^^39^^,^^40^ The products used at this time contained high levels of neutralizing anti-SARS-CoV-2 Spike antibody and were given at high dose.^12^ However, persistence of SARS-CoV-2 and continued evolution has been observed after IVIG administration (albeit of an unknown dose and duration),^41^ and theoretically this may become a greater concern if antibody titers wane over time in the donor population.

Our analysis revealed that most fixed spike mutations emerging in this chronically infected patient are likely to reduce viral fitness as they are rare in global sequence databases. Structural studies indicate two of the mutations arising,T573I and A1078V, may destabilize the structure of the spike protein.^10^^,^^42^

Long-term persistent infection did, however, induce a high magnitude, broadly targeting, proliferative, and functional memory T cell response. SARS-CoV-2-specific T cell memory persisted up to 2 years after viral control at levels that are higher than those seen in immunocompetent patients 4 months after Wuhan hu-1 infection.^22^^,^^28^ Interestingly, rapid production of multiple antiviral cytokines and good proliferative capacity of the generated T cell memory shows that T cells retained *in vitro* responsiveness despite persistent antigen exposure for 2.5-year. Viruses that have evolved to allow chronic infection in humans can alter the immune landscape during infection and drive exhaustion via multiple mechanisms^26^^,^^43^^,^^44^^,^^45^; however, antigen exposure alone does not always drive exhaustion, as is observed in autoimmunity and latent infections such as EBV and CMV, which could explain the lack of exhaustion here. Phenotypic analysis showed that despite polyfunctional cytokine response, activation marker induction, and proliferation in response to peptide stimulation, spike-specific memory T cells had upregulated coinhibitory receptor expression. Repeated antigen exposure had changed the T cell memory phenotype and potentially *in vivo* responsiveness, but it fell short of the *in vitro* exhaustion/non-responsiveness seen after chronic viral infections with hepatitis B and C.^43^^,^^44^^,^^46^

The expansion of a large, spike-dominated T cell response is characteristic of more severe SARS-CoV-2 infections; it has been suggested that this skewing toward a spike-specific immune response is the result of higher and longer antigen exposure.^47^^,^^48^ Even in mild infections, those with the lowest Ct value by PCR (i.e., highest viral load) tend to generate a more spike-dominated T cell response.^22^ T cell responses recognizing the NSPs of the core RTC have been associated with early viral control and protection from detectable infection.^22^ A detectable response to these proteins was observed in our patient despite failure to clear the virus; however, the ratio of RTC:Spike T cells was lower than that observed in acute-resolving infection, showing that they remain subdominant. The RTC proteins in ORF1ab are highly conserved, likely due to functional constraint and their importance in the viral life cycle.^22^^,^^23^ Despite ORF1ab constituting more than two-thirds of the overall SARS-CoV-2 genome by length, only 45% of the mutations arising in our patient were within ORF1ab, suggesting that they acquired mutation more slowly than other ORFs. The exact impact of mutations in ORF1ab is hard to determine due to a lack of tractable models to study them *in vitro*, unlike for receptor binding and antibody evasion by changes in spike.

At the population level, the diversity of HLA molecules ensures that each individual recognizes a different set of epitopes across a virus, which spreads the selection pressure and makes it more difficult for the virus to accrue T cell escape mutations. This also makes it difficult to see positive selection at T cell epitopes. For influenza, positive selection can be observed by comparing evolution at HLA presented epitopes isolated in parallel in humans and pigs (where there is no presentation and selection pressure).^49^ Long-term T cell escape occurs over decades^50^ and in mouse models escape mutations revert back to wild type in a host lacking the HLA to present the escaped epitope.^51^ Intra-host escape from T cells is, however, well described for viruses that commonly cause chronic infections, such as HIV, and hepatitis B and C.^44^^,^^52^^,^^53^ The selection pressure from T cell immunity persists throughout the infection at the specific epitopes presented by the patient’s HLA, which can drive the fixation of mutations in known T cell epitopes. Here, 69% of the CD8 epitopes in which mutations fell were expected to be negatively impacted by *in silico* prediction of peptide MHC binding and immunogenicity. Most importantly, we have shown that mutations arising within both CD4 and CD8 epitopes for which T cell responses are seen, result in lack of recognition when T cells are stimulated with the emerging variant sequences, demonstrating functional T cell escape. It was unfortunately not possible to test all potential epitopes that could have been restricted by the patients HLA to definitively confirm the extent of T cell escape, however, when assessing just a small number of immunodominant epitopes loss of functional recognition and escape due to viral mutation relative to ancestral B.31 sequence was observed.

The magnitude of the T cell response to these 12 epitopes is, however, low and just a fraction of the total T cell response in this patient. Many epitopes were retained despite persistent infection, and the resulting mutant virus had lost a small subset of the total epitopes seen across the SARS-COV-2 genome. Individual peptide responses were more readily detectable for CD4 responses and only 1 of the two CD8 epitopes showed escape, versus 6/7 CD4 responses. A complicated interplay between which epitopes are best processed and presented to T cells, which are functionally constrained, and therefore unable to accept mutations without affecting viral replication, and which epitopes can be recognized by the T cell receptors (TCRs) present in a person’s repertoire contribute to which epitopes are likely to escape during an infection.

Lineage defining mutations for SARS-CoV-2 variants impact just a small number of epitopes and lead to a small but consistent drop in T cell recognition.^54^ We have previously shown that despite most lineage defining mutations falling in T cell epitopes, this is not occurring more than by chance, suggesting that selection pressure by T cells is unlikely the main driver of evolution of these lineage defining mutations.^54^ As with the mutations arising in emerging variants,^55^^,^^56^ the mutations arising during this persistent infection affected just a subset of epitopes, leading to a small but detectable loss of T cell recognition. This suggests that persistent infection offers a window into the future of potential escape mechanisms seen in variants of concern.

It is not possible to determine whether the T cell response in this patient was responsible for keeping viral replication below that which is detectable for the majority of the infection, and/or whether T cell-driven inflammation is responsible for the ongoing lung pathology observed. In postmortem analysis of persistent infections, lung pathology at the time of death was similar to that seen at the time of PCR positivity in acute SARS-CoV-2 infection and nucleoprotein and spike antigens were readily detectable.^32^ Additionally, persistent infections in immuno-competent individuals have been linked to higher rates of Long-COVID^17^; a better understanding of immune-surveillance and mechanisms of immune escape in persistent infections may shed light on the pathophysiology of Long-COVID and highlight therapeutic targets, as well as informing clinical management of immunocompromised individuals.

### Limitations of the study

This study may have limited generalizability due to the fact that it is a single case report. The absence of detectable virus in samples collected throughout the 2.5-year infection period prevents a detailed analysis of the evolutionary trajectory. Although the GISAID database represents the most extensive collection of viral sequences over time and geographical regions, a major caveat when comparing the frequency of mutations is that there is inherent sampling bias due to uneven sequencing efforts. Additionally, blood samples for immunological assessment were taken more than 1 year after resolution of the infection, which may have affected the T cell responses we detected. Our investigation covered only a subset of T cell epitopes affected by viral mutations, providing an incomplete picture of the total immune escape landscape.

Overall, the viral evolutionary pathways in immunocompromised patients highlight the potential for SARS-CoV-2 to explore a wider mutational landscape in the absence of full immune constraint. However, without significant humoral immune escape mutations, it is likely that antibody-based treatment (i.e., IVIG, convalescent plasma, or effective monoclonal antibodies) would be effective in these patients in combination with antivirals. This work highlights the importance of close monitoring immunocompromised patients, where infections may not always be detectable by nasal swab and Real-time PCR, but where therapeutic intervention may be required to limit progression of lung pathology. While this study describes a single infection, the findings suggest that T cell-mediated selective pressure may be selecting epitope variants that confer immune evasion. Larger studies will be required to fully elucidate the prevalence and phenotypic consequences of this phenomenon. Nonetheless, our work highlights the potential importance of monitoring T cell reactivity against mutated epitopes over time, as changes in epitope recognition may serve as an early indicator of viral immune escape. Integrating longitudinal genomic and immunologic data from larger cohorts promises to shed further light on the interplay between the human immune system and evolving viral genomes.

## Resource availability

### Lead contact

Requests for further information and resources should be directed to and will be fulfilled by the lead contact, Leo Swadling (l.swadling@ucl.ac.uk).

### Materials availability

This study did not generate new unique reagents.

### Data and code availability

#### Data availability

Data reported in this paper will be shared by the lead contact upon request.

Viral sequences are available at: Day 899 of infection: Zenodo: https://zenodo.org/records/17341064 and GISAID, hCoV-19/England/22K457422/2022.

Viral sequence of subsequent infection in October 2024 with XEC.2 strain—European Nucleotide Archive (ebi.ac.uk/ena): Biosample: SAMEA117560877.

#### Code availability

This paper does not report original code.

#### All other items

Any additional information required to reanalyze the data reported in this paper are available from the lead contact upon request.

## Acknowledgments

We thank the patient and their family for participation in this study and all the clinical staff who helped with recruitment and sample collection; Dr. Marina Zamudio Escalera for feedback on the manuscript; Ryan Hisner for advice on sequencing data. UCL IIT FACS facility for assistance with flow cytometry assays; UCL genomics for assistance with sequencing; members of all of the contributing and submitting laboratories around the globe who have openly shared large numbers of UK SARS-CoV-2 assemblies via GISAID. L.S. is funded by a Rosetrees Trust and Pears Foundation Advancement fellowship and Wellcome Trust Career Development Award ref. 302473/Z/23/Z. R.M. is funded by an MRC DTP PhD studentship (MR/W006774/1). S.C. is funded by a BBSRC LIDo-DTP studentship (BB/T008709/1). K.T. is funded by an MRC Clinical Research Training fellowship (MR/Y00146X/1). J.B. is an NIHR Senior Investigator and receives funding from the UCL/UCLKBackspaceH NIHR Biomedical Research Center.

## Author contributions

D.L., J.B., and L.S. conceived the project and obtained funding; J.A.G.A., R.M., K.T., S.C., L.F., J.T., T.T., L.S., and D.L. collected and/or processed samples; J.A.G.A. performed sequencing and J.A.G.A. and S.C. performed sequence analysis; K.T. and D.L. provided clinical data and interpretation; R.M., L.F., J.T., and L.S. performed immunological assays and analysis; T.T. and N.M. performed and analyzed HLA typing; J.A.G.A., R.M., L.S., J.B., and D.L. analyzed and interpreted the data; D.L., J.A.G.A., and L.S. prepared the manuscript. All authors reviewed and approved the manuscript.

## Declaration of interests

J.B. has received consultancy fees (paid to institution) from GSK, Moderna, HVivo, and Symbios. D.L. has received research grants from GSK and Bristol Myers Squibb, consultancy fees from GSK (paid to institution), speaker fees from Biotest, Takeda, AstraZeneca, and Roche, advisory board fees from CSL Behring and support to attend a conference from Octapharma. All other authors declare no competing interests.

## STAR★Methods

### Key resources table

**Table:** 

REAGENT or RESOURCE	SOURCE	IDENTIFIER
**Antibodies**		

ELISpot biotinylated IFNγ detection antibodies clone 7-B6-1	Mabtech	3420-6
ELISpot coating antibody human anti-IFNγ clone D1K	Mabtech	3420-3
goat anti-biotin alkaline phosphatase	Vector Laboratories	SP-3020
anti-human IL-2 PerCp-eFluor710 clone MQ1-17H12	Invitrogen	46-7029-42
anti-human TNFα FITC clone MAb11	BD Bioscience	554512
anti-human CD8α BV785 clone RPA-T8	BioLegend	301046
anti-human IFNγ BV605 clone B27	BD Bioscience	502536
anti-human IFNγ APC clone 4S.B3	BioLegend	502512
anti-human CD3 BUV805 clone UCHT1	BD Bioscience	612895
anti-human CD4 BUV395 clone SK3	BD Bioscience	563550
anti-human CD154 (CD40L) Pe-Cy7 clone 24-31	BioLegend	310832
anti-human CD3 for plate coating and stimulation clone OKT3	ThermoFisher Scientific	16-0037-85
soluble anti-CD28 antibodies	ThermoFisher Scientific	16-0289-85
anti-human CD8-BUV496 clone RPA-T8	BD biosciences	612942
anti-human CD56-BUV737 clone NCAM16.2	BD biosciences	612766
anti-human CD127-BV510 clone AO19D5	BioLegend	351332
anti-human PD-1-BV750 clone EH12.2H7	BioLegend	329966
anti-human Tim3-BB515 clone 7D3	BD biosciences	565568
anti-human OX40-BUV661 clone L106	BD biosciences	750526
anti-human CD4-SB550 clone SK3	BioLegend	344656
anti-human 4-1BB/CD137-BV421 clone 4B4-1	BioLegend	309820
anti-human Lag-3/CD223-Pe-Cy7 clone 3DS223H	Invitrogen	25-2239-41
anti-human CD69-APC-Cy7 clone FN50	BD biosciences	310913
anti-human CTLA-4-BV605 clone BNI3	BioLegend	369610
anti-human Tox-PE clone REA473	Miltenyi Biotec	130-120-716

**Biological samples**		

PBMC: Persistent infection	This paper	NHS Research Ethics Committee approved protocol (04/Q0501/119)
PBMC: Health care worker cohort for comparator data	COVIDsortium bioresource	UK National Research Ethics Service (20/SC/0149) and registered at https://ClinicalTrials.gov (NCT04318314)
PBMC: Pre-pandemic healthy donors	This paper	NHS Research Ethics Committee number 11/LO/0421

**Chemicals, peptides, and recombinant proteins**		

Pancoll	Pan Biotech	P04-60500
FCS	Hyclone / Merck	F2442
DMSO	Sigma-Aldrich	D2650-100ml
Overlapping peptides coving SARS-CoV-2 proteins	GL biochem	Custom synthesis. Peptide lists in: (refs.^22^^,^^28^^,^^57^).
Peptide Pool, Cytomegalovirus, Epstein-Barr Virus and Influenza Virus (FEC)	NIH	ARP-9808
SARS-CoV-2 peptide array, Gene S	NIH	NR-52402
RPMI	Life Technologies	21875091
100× penicillin and streptomycin solution	Life Technologies	15140122
Elispot Plates	Merck-Millipore	MSIP4510
DNAse	Sigma-Aldrich	11284932001
Concavalin A	Sigma-Aldrich	C2010
PBS	Life Technologies	14190169
BSA	Sigma-Aldrich	A7030-10G
BCIP/NBT Phosphatase Substrate	ThermoFisher Scientific	34042
Cell Trace Violet	ThermoFisher Scientific	C34571
recombinant human IL-2	peprotech	200-02-1mg
brefeldin A	Sigma-Aldrich	B6542-5MG
fixable live/dead Near infrared	Invitrogen	L10119
fixable blue dead cell stain kit	Invitrogen	L23105
TF staining Buffer kit	eBiosciences	00-5523-00
Brilliant Stain Buffer	BD biosciences	566349

**Deposited data**		

Viral Sequence Day 899 of infection	This paper	Zenodo:17341064 https://zenodo.org/records/17341064
Viral Sequence Day 899 of infection	This paper	GISAID: hCoV-19/England/22K457422/2022
Viral sequence of subsequent infection in October 2024 with XEC.2 strain	This paper	European Nucleotide Archive (ebi.ac.uk/ena): Biosample: SAMEA117560877.

**Software and algorithms**		

fastp	Chen et al.^58^	github.com/OpenGene/fastp
iVAR	Grubaugh et al.^59^	github.com/andersen-lab/ivar
Phylogenetic Assignment of Named Global Outbreak LINeages (PANGOLIN)	O'Toole et al.^60^	github.com/cov-lineages/pangolin
Ultrafast Sample placement on Existing tRees (UShER)	Turakhia et al.^13^	github.com/yatisht/usher
GISAID sequence database	Gangavarapu et al.^15^	gisaid.org/
NCBI Virus database	NCBI^61^	www.ncbi.nlm.nih.gov/labs/virus/vssi/#/
MAFFT Multiple Sequence Alignment Software	Katoh et al.^62^	mafft.cbrc.jp/alignment/software/
IQ-Tree	Nguyen et al.^63^	iqtree.github.io/
ape 5.0	Paradis et al.^64^	cran.r-project.org/web/packages/ape/index
Nextstrain	Hadfield et al.^65^	nextstrain.org/
Immune Epitope Database (IEDB)	Vita et al.^66^	www.iedb.org/
NetMHCpan	Reynisson et al.^67^	services.healthtech.dtu.dk/services/NetMHCpan-4.1/
NetMHCIIpan	Reynisson et al.^67^	services.healthtech.dtu.dk/services/NetMHCIIpan-4.1/
SPICE (v.6.0) and pestle (v.2.0),	Roederer et al.^68^	niaid.github.io/spice/

**Other**		

Stanford Coronavirus Resistance Database	Tzou et al.^24^	covdb.stanford.edu/

### Experimental model and study participant details

#### Samples for immune assays and isolation of PBMC

Samples from the persistently affected patient were collected under an NHS Research Ethics Committee approved protocol (04/Q0501/119) and the patient provided written, informed consent. Blood was taken in December 2023 and Nov 2024, 12 months and 22 months post-resolution of the chronic infection. T cell memory responses persisting after infection were assayed.

Peripheral blood mononuclear cells were isolated from heparinised blood samples using Pancoll (Pan Biotech) density-gradient centrifugation. Isolated PBMCs were cryopreserved in fetal calf serum (FCS) containing 10% DMSO and stored in liquid nitrogen. All T cell assays reported here were performed on cryopreserved PBMCs.

Comparator data (Figures 3C–3F) on T cell responses measured from samples taken pre-pandemic and as part of the COVIDsortium bioresource during the first wave of SARS-CoV-2 infections (March-August 2020) were previously published in.^22^^,^^28^ The COVIDsortium bioresource was approved by the ethical committee of UK National Research Ethics Service (20/SC/0149) and registered at https://ClinicalTrials.gov (NCT04318314). Full study details of the bioresource (participant screening, study design, sample collection and sample processing) have previously been described.^69^ Pre-pandemic samples from healthy donors were collected and cryopreserved before August 2019 under NHS Research Ethics Committee number 11/LO/0421. All participants provided written informed consent and the study conformed to the principles of the Helsinki Declaration.

#### Sample processing

To monitor SARS-CoV-2 infection status over time, serial nasopharyngeal swabs were collected and tested using quantitative PCR (qPCR). A cycle threshold value ≤ 40 was considered positive for SARS-CoV-2 RNA. Samples with a positive result were sent for sequencing to determine sample lineage as previously described.^12^

### Method details

#### Sequencing

Libraries were hybridized to a biotinylated RNA bait set (Agilent Technologies) designed against the SARS-CoV-2 reference genome (NCBI, National Center for Biotechnology Information, GenBank MN908947.3) using the Agilent SureSelectXT Automated Target Enrichment Protocol. Captured libraries were indexed and processed using the Bravo Automated Liquid Handling Platform (Agilent). All libraries were sequenced on a MiSeq genome sequencer (Illumina) with 2×150 bp paired end reads at a target depth of 5000x coverage per genome. Positive (SARS-CoV-2 Alpha Variant RNA) and negative (nuclease-free water) controls were included in each batch of samples starting from RNA extraction through sequencing to monitor contamination.

#### Sequence analysis

Raw fastq sequencing reads were processed using fastp^58^ for adapter trimming and quality filtering using default parameters. Reads were mapped to the Wuhan-Hu-1 SARS-CoV-2 reference genome (GenBank accession MN908947.3) and the primer binding sites were trimmed from the alignment based on the ARTIC v4.1 primer scheme coordinates. Variants were called using iVAR^59^ with the default settings requiring ≥10X coverage depth. Only genomes with ≥90% coverage of the reference at 10X depth were retained for analysis. The Phylogenetic Assignment of Named Global Outbreak LINeages (PANGOLIN) SARS-CoV-2 tool^60^ was used to assess the lineage of each sample. The entire workflow from raw reads to consensus assembly and lineage typing was processed through the nf-core/viralrecon v2.6.0 pipeline.

#### Sample placement analysis

Global sample placement analysis was performed using UShER,^13^ which is a tool for inserting query sequences into a fixed reference phylogeny to determine evolutionary relationships. This was used to place the sequenced SARS-CoV-2 samples in global context using more than 15M sequences publicly available as of July 2023, which represent global SARS-CoV-2 diversity. Outbreak.info was used to assess the frequency of individual mutations and B.31 lineage within the GISAID sequence database.^15^

#### Phylogenetic analysis

SARS-CoV-2 genome sequences were downloaded from the NCBI Virus database^61^ (23rd October 2025). Only complete human-derived genomes were included, with a minimum length of 27,000 nucleotides, no ambiguous characters, and exclusion of vaccine strains, laboratory-passaged isolates, and environmental samples. Up to five sequences per country were randomly sampled for each period (January 1 – December 31) to ensure geographic diversity, grouped by collection year as 2020 (n = 364), 2021 (n = 361), and 2022 (n = 271). Duplicate sequences were removed before merging, and the viral sequence identified from the patient (chronic sequence) was added to yield 997 genomes in total. All sequences were aligned using MAFFT^62^(v7.526) with the FFT-NS-2 progressive algorithm, and a maximum-likelihood tree was inferred with IQ-TREE^63^(v2.3.6) under the GTR+G model using 1,000 SH-aLRT replicates. The Wuhan-Hu-1 reference genome (NCBI Virus accession NC_045512.2) was included as an outgroup to root the tree, and tips were coloured by year of collection. Pairwise genetic (p-)distances between the chronic sequence and every other genome were calculated in R^70^ (v4.3.3) using the “ape” package,^64^ defined as the proportion of nucleotide sites differing after excluding gaps and ambiguous bases. Genetic distances and absolute mutation counts were compared between year groups using separate pairwise Wilcoxon rank-sum tests with Benjamini–Hochberg correction to control for false positives arising from multiple comparisons.

#### Novel variant analysis

In the absence of samples from the early stages of infection, the programs augur and auspice within Nextstrain^65^ were then used to analyse and visualize the locations of mutations arising in the sequenced samples compared to the closest publicly available sequence. The number and positions of SNPs and indels unique to each sample were assessed to identify *de novo* mutations potentially arising during infection.

#### Antibody immune escape and drug resistance

The Stanford Coronavirus Resistance Database (CoV-RDB)^24^ contains curated published data on SARS-CoV-2 neutralization susceptibility profiles for variants and individual spike mutations to monoclonal antibodies (493 mAb resistant mutations described), convalescent plasma, and vaccinee sera, as well as RdRP (RNA Dependent RNA Polymerase; 19 resistance mutations identified) and 3C-like protease (3CLpro; 113 resistance mutations identified) mutations involved in drug resistance (Table S8). The database was queried using the list of mutations observed at day 899 of persistent infection to identify If any had been previously associated with resistance to nAb, 3CLpro or RdRP inhibitors.

#### HLA typing

HLA typing was performed at Anthony Nolan, a European Federation for Immunogenetics (EFI) and UKAS:ISO15189 accredited HLA typing laboratory. Briefly, genomic DNA underwent PCR amplification for each gene individually using in-house primers and protocols. Samples were indexed and underwent library preparation for sequencing using the GenDx NGSgo Library Kit according to the manufacturer’s protocols (GenDx, Utrecht, The Netherlands). Sequencing was performed on a MiSeq instrument (Illumina, San Diego, CA, USA). Sample identification and HLA typing assignment were performed using NGS Engine (GenDx) and IPD/IMGT-HLA Database v3.57.

#### Impact of non-synonymous mutations on peptide presentation to T cells

To evaluate potential T cell immune escape mutations, experimentally validated epitopes described in Grifoni et al.^20^ and Immune Epitope Database (IEDB^66^) which contain non-synonymous mutations arising during the persistent infection were identified and listed in Tables S3 and S4 for CD4 and CD8 epitopes respectively.

Where mutations fell within validated epitopes, the ancestral sequence peptide and variant peptide were compared for the likelihood that they would be naturally presented on MHC (elution rank %; rank is relative to a set of random peptides). NetMHCpan-4.1 and NetMHCIIpan-4.1 were used to predict the elution scores for peptides on MHC class I and II alleles respectively,^67^ using default parameters. For validated CD8 epitopes without a known HLA-restriction, EL scores (Table S4) can be calculated for a set of HLA supertype representatives on NetMHCpan 4.1. Where these predictions indicated a probably weak or strong binder, ‘predicted’ is given next to the HLA allele in (Table S4). There is no equivalent representative list of class II supertypes.

For MHC class I binding, a weak binder is classed as a peptide with an EL rank <2.0% and a strong binder <0.5% rank. For MHC class II a weak binder is classed as a peptide with an EL rank <5.0% and a strong binder <1.0% rank.^67^ For validated epitopes without a known HLA-restriction, EL scores can be calculated for a set of HLA supertype representatives on NetMHCpan 4.1. Where these predictions indicated a weak or strong binder, ‘predicted’ is given next to the HLA allele.

Where a mutation leads to a variant peptide with a lower predicted EL rank and HLA binding category (a strong binding peptide becoming a weak binder or unclassified, or a weak binder becoming unclassified), this is considered a negative impact on presentation and potential immune escape. Mutations can also lead to a higher binding rank which indicates they are predicted to improve peptide binding to HLA.

Variant peptides that were used in immunoassays in Figure 5 are highlighted in green. E, envelope; EL, elution rank; M, membrane; NP, nucleoprotein; NSP, non-structural protein; ORF, open-reading frame; S, spike; SB, strong binder; WB, weak binder. Immunodominant was defined as being recognized in 3 or more studies in the meta-analysis of validated epitopes in.^20^

#### *In silico* prediction of immunogenicity for MHC class I epitopes

Where mutations fell within validated epitopes, the ancestral sequence peptide and variant peptide sequences at epitopes were compared for predicted immunogenicity using a simple model available at www.iedb.org.^71^ Amino acids at positions 1, 2, and C-terminus amino acid were masked and not considered for immunogenicity scores. Scores greater than 0 indicate the peptide is more likely than not to elicit an immune response, while a score less than 0 indicates the inverse.

#### Peptides

Full lists of the peptides contained in pools of overlapping peptides covering structural^57^ (see below for details of Spike peptides) and RTC^22^^,^^28^ proteins have previously been described (15-mer peptides overlapping by 10 amino acids, GL Biochem Shanghai, >80% purity). A list of optimal peptides corresponding to the ancestral or variant sequence (corresponding to non-synonymous mutations seen at day 899 of a persistent infection) is given in Table S7. Optimal length peptides were purchased at >95% purity, GL Biochem Shanghai.

The following reagents were obtained through the NIH HIV Reagent Program, Division of AIDS, NIAID, NIH: Peptide Pool, Cytomegalovirus, Epstein-Barr Virus and Influenza Virus (FEC) Control, ARP-9808, and SARS-CoV-2 peptide array, Gene S NR-52402, contributed by DAIDS, NIAID.

#### IFNγ ELISpot

The IFNγ ELISpot assay was performed as previously described on cryopreserved PBMCs.^22^ Culture medium for human PBMCs (R10) was sterile 0.22-μm-filtered RPMI medium (Thermo Fisher Scientific) supplemented with 10% by volume heat-inactivated (1 h, 64 °C) FCS (Hyclone) and 1% by volume 100× penicillin and streptomycin solution (Gibco-BRL).

ELISpot plates (Merck-Millipore, MSIP4510) were coated with human anti-IFNγ antibodies (1-D1K, Mabtech; 10 μg/ml) in PBS overnight at 4 °C. The plates were washed six times with sterile PBS and blocked with R10 for 2 h at 37 °C with 5% CO_2_. PBMCs were thawed and rested in R10 with DNAse for 3 h at 37 °C with 5% CO_2_ before being counted to ensure that only viable cells were included. PBMCs (400,000 per well) were seeded in R10 and were stimulated for 16–20 h with SARS-CoV-2 peptide pools (2 μg/ml per peptide) at 37 °C in a humidified atmosphere with 5% CO_2_.

Internal plate controls were two DMSO wells (negative controls), concanavalin A (ConA, positive control; Sigma-Aldrich) and FEC (HLA I-restricted peptides from influenza, Epstein–Barr virus and CMV; 1 μg/ml per peptide). ELISpot plates were developed with human biotinylated IFNγ detection antibodies (7-B6-1, Mabtech; 1 μg/ml) for 3 h at room temperature, followed by incubation with goat anti-biotin alkaline phosphatase (Vector Laboratories; 1:1,000) for 2 h at room temperature, both diluted in PBS with 0.5% BSA by volume (Sigma-Aldrich), and finally with 50 μl per well of sterile filtered BCIP/NBT Phosphatase Substrate (Thermo Fisher Scientific) for 7 min at room temperature. Plates were washed in double-distilled H_2_O and left to dry overnight before being read on the AID classic ELISpot plate reader (Immunospot S6 Universal M2 ELISpot Reader). Plate imaging and count settings were automated and consistent across all experiments.

The average of two DMSO wells was subtracted from all peptide-stimulated wells for a given PBMC sample and any response that was lower in magnitude than 2 standard deviations of these sample-specific DMSO control wells was not considered to be a peptide-specific response (assigned value 0). Results were expressed as IFNγ SFCs per 10^6^ PBMCs after background subtraction.

#### Short-term T cell peptide stimulations

Short-term peptide stimulations were performed as described previously^22^ and were used to expand T cell responses that were low magnitude *ex vivo*, so that cross-recognition of ancestral and variant peptides could be assessed.

Frozen PBMCs were thawed and washed twice with sterile PBS. When cell trace violet (CTV) staining was performed, PBMC were resuspended in PBS (2–10 × 10^6^ PBMCs) and 0.5 μl of 5 mM stock CTV (Thermo Fisher Scientific) was added per sample with mixing. PBMCs were stained in the dark for 10 min at 37 °C in a humidified atmosphere with 5% CO2. Ten-times volume of cold R10 was added to stop the staining reaction, and cells were incubated for 5 min on ice. Cells were washed in PBS and incubated for 5 min at 37 °C before being transferred to a new tube and were washed again in R10.

When CTV staining was not measured alongside T cell expansion, PBMC were plated in 96-well plates (2–4 × 10^5^ PBMCs in 200 μl R10) and stimulated with peptide pools (2 μg/ml per peptide) for 5-10 days (5 days for peptide pools, 8-10 days for individual peptide stimulations) in R10 supplemented with 0.5 μg/ml soluble anti-CD28 antibodies (Thermo Fisher scientific) and 20 U/ml recombinant human IL-2 (Peprotech). Then, 100 μl medium was added on day 1, and 100 μl medium was removed and replaced with R10 supplemented with anti-CD28 and IL-2 as above on days 3 and 6. On the penultimate day of culture PBMCs were restimulated with peptide pools (2 μg/ml per peptide) and brefeldin A (10 μg/ml; Sigma-Aldrich). After 16–18 h restimulation, PBMCs were collected, washed in PBS and stained with fixable live/dead at 4°C (Near infrared, Thermo Fisher Scientific, 1:1,000), washed in PBS, before being fixed in fix/perm buffer (TF staining buffer kit, eBioscience) for 20 min at room temperature. Cells were washed in PBS and incubated in perm buffer (TF staining buffer kit, diluted 1:10 in double-distilled H2O) for 20 min at room temperature, washed in PBS and resuspended in perm buffer with saturating concentrations of anti-human antibodies for intracellular staining: anti-IL-2 PerCp-eFluor710 (Invitrogen, MQ1-17H12, 1:50), anti-TNFα FITC (BD Bioscience, MAb11, 1:100), anti-CD8α BV785 (BioLegend, RPA-T8, 1:200), anti-IFNγ BV605 (BD Biosciences, B27, 1:100), anti-IFNγ APC (BioLegend, 4S.B3, 1:50), anti-CD3 BUV805 (BD Biosciences, UCHT1, 1:200), anti-CD4 BUV395 (BD Biosciences, SK3, 1:200), and anti-CD154 (CD40L) Pe-Cy7 (BioLegend, 24-31, 1:50). Cells were washed twice in PBS and analysed using the BD LSRII flow cytometer. Cytometer voltages were consistent across batches. Fluorescence minus one (FMOs) and unstimulated samples were used to determine gates applied across samples. Data were analysed using FlowJo v.10.7 (TreeStar). Example gating shown in Figure S4.

CTV dilution and/or staining with anti-human-IFNγ and anti-human-TNF antibodies were used to identify antigen-specific T cells. An unstimulated control well (equivalent DMSO volume to peptide wells) was included for each PBMC sample and the percentage of responding CD4 or CD8 cells in wells not stimulated with peptide were subtracted as background cytokine release from all peptide-stimulated wells.

Polyfunctionality, defined as the number of cytokines co-produced by T cells after expansion for 10 days, was assessed using SPICE (v.6.0) and pestle (v.2.0), available at GitHub (https://niaid.github.io/spice/; Figures 4C and 4D^68^).

Boolean gating was used to identify the percentage of T cells making possible combinations of the following cytokines: IFNγ, TNF, IL-2, or CD154. Pestle was used to background-subtract the percentage of cytokine-producing cells from unstimulated wells that were run in parallel and to format data for visualization in SPICE. The proportion of T cells making a specific number of cytokines in combination is presented as pie graphs (base mean) and pie arcs represent the proportion making a given cytokine.

#### Activation-induced marker assay

PBMC were stimulated with Spike (Wuhan hu-1; NIH) and FEC peptide pools (2 μg/ml per peptide) or plate bound anti-CD3 with soluble anti-CD28 (10 ug/ml, Invitrogen) in 96-well U bottom plates at 1x10^6^ cells per well. A stimulation with an equimolar amount of DMSO was performed as negative control. PBMC were collected 22 hours post-peptide stimulation and washed with PBS. Cells were then stained with live/dead fixable blue dead cell stain kit (Invitrogen, L23105, 1:1000 PBS) for 20 minutes at 4 °C, before being washed with PBS then resuspended in a 1:1 mixture of PBS:BD Brilliant stain buffer (BD biosciences) with surface panel antibodies for 30 minutes at 4°C: CD3-BUV395 (BD Biosciences, UCHT1, 1:100), CD8-BUV496 (BD biosciences, RPA-T8, 1:400), CD56-BUV737 (BD biosciences, NCAM16.2, 1:200), CD127-BV510 (Biolegend, AO19D5, 1:50), PD-1-BV750 (Biolegend, EH12.2H7, 1:200), Tim3-BB515 (BD biosciences, 7D3, 1:100), OX40-BUV661 (BD biosciences, L106, 1:50), CD4-SB550 (Biolegend, SK3, 1:400), 4-1BB/CD137-BV421 (Biolegend, 4B4-1, 1:100), Lag-3/CD223-Pe-Cy7 (Biolegend, 7H2C65, 1:50), CD69-APC-Cy7 (BD biosciences, FN50, 1:400). Cells were washed with PBS, then fixed for 1 hour at 4 °C using a TF staining buffer set (eBioscience). Following washing with perm buffer (eBioscience 1:10 dilution in ddH20), cells were resuspended in perm buffer with intracellular panel antibodies for 30 minutes at 4°C: CTLA-1-BV605 (Biolegend, BNI3, 1:200), Tox-PE (Miltenyi Biotec, REA473, 1:50). Cells were washed twice with perm buffer before being resuspended in PBS and acquired on the Cytek Aurora.

Stimulation responsive T cells were identified as those upregulating the activation markers CD69, 4-1BB and OX40^72^ (AIM+ defined as OX40+CD69^+^ for CD4 T cells, 4-1BB+ and CD69^+^ for CD8 T cells). The magnitude of the stimulation-specific response was calculated by subtracting the AIM+ population in the DMSO negative control for the donor and time point matched sample.

### Quantification and statistical analysis

Data were assumed to have a non-Gaussian distribution, and nonparametric tests were used throughout. For single-paired and unpaired comparisons, Wilcoxon matched-pairs signed-rank tests and Mann-Whitney U-tests were used. For multiple unpaired comparisons, Kruskal–Wallis one-way ANOVA with Dunn’s correction was used. For correlations, Spearman’s r test was used. P < 0.05 (two-tailed) was considered to be significant. Prism v.10.4.1 for Mac was used for analysis. Details are provided in the figure legends. ∗P ≤ 0.05; ∗∗P ≤ 0.01; ∗∗∗P ≤ 0.001; ∗∗∗∗P < 0.0001. Only statistically significant results are reported in figures.

## Supplemental information

Document S1. Figures S1–S6

Table S1. Negative investigational infection screens during suspected persistent SARS-CoV-2 infection

Table S2. Sequencing quality control metrics for SARS-CoV-2 samples

Table S3. List of validated CD4 T cell epitopes in which mutations arising during persistent infection fall and predicted impact on peptide HLA binding

Table S4. List of validated CD8 epitopes in which mutations arising during persistent infection fall and predicted impact on peptide HLA binding

Table S5. HLA type of patient experiencing persistent infection

Table S6. Predicted impact of mutations on peptide immunogenicity

Table S7. Peptides used in immune assays

Table S8. Lists of mutations associated with resistance to mAb, protease inhibitors or RNA-dependent RNA polymerase inhibitors taken from Stanford coronavirus database (CoV-RDB)

## References

[1] Chen N., Zhou M., Dong X., Qu J., Gong F., Han Y., Qiu Y., Wang J., Liu Y., Wei Y. (2020). Epidemiological and clinical characteristics of 99 cases of 2019 novel coronavirus pneumonia in Wuhan, China: a descriptive study. Lancet.

[2] Vaid N., Ardissino M., Reed T.A.N., Goodall J., Utting P., Miscampbell M., Condurache D.G., Cohen D.L. (2021). Clinical characteristics and outcomes of immunosuppressed patients hospitalized with COVID-19: experience from London. J. Intern. Med..

[3] Choi B., Choudhary M.C., Regan J., Sparks J.A., Padera R.F., Qiu X., Solomon I.H., Kuo H.H., Boucau J., Bowman K. (2020). Persistence and Evolution of SARS-CoV-2 in an Immunocompromised Host. N. Engl. J. Med..

[4] Stanevich O.V. (2023). SARS-CoV-2 escape from cytotoxic T cells during long-term COVID-19. Nat. Commun..

[5] Buckland M.S., Galloway J.B., Fhogartaigh C.N., Meredith L., Provine N.M., Bloor S., Ogbe A., Zelek W.M., Smielewska A., Yakovleva A. (2020). Treatment of COVID-19 with remdesivir in the absence of humoral immunity: a case report. Nat. Commun..

[6] Weigang S., Fuchs J., Zimmer G., Schnepf D., Kern L., Beer J., Luxenburger H., Ankerhold J., Falcone V., Kemming J. (2021). Within-host evolution of SARS-CoV-2 in an immunosuppressed COVID-19 patient as a source of immune escape variants. Nat. Commun..

[7] Khatamzas E., Antwerpen M.H., Rehn A., Graf A., Hellmuth J.C., Hollaus A., Mohr A.W., Gaitzsch E., Weiglein T., Georgi E. (2022). Accumulation of mutations in antibody and CD8 T cell epitopes in a B cell depleted lymphoma patient with chronic SARS-CoV-2 infection. Nat. Commun..

[8] Willett B.J., Grove J., MacLean O.A., Wilkie C., De Lorenzo G., Furnon W., Cantoni D., Scott S., Logan N., Ashraf S. (2022). SARS-CoV-2 Omicron is an immune escape variant with an altered cell entry pathway. Nat. Microbiol..

[9] Escalera-Zamudio M., Tan C.C.S., van Dorp L., Balloux F. (2024). Early evolution of BA.2.86 sheds light on the origins of highly divergent SARS-CoV-2 lineages. bioRxiv.

[10] Harari S., Tahor M., Rutsinsky N., Meijer S., Miller D., Henig O., Halutz O., Levytskyi K., Ben-Ami R., Adler A. (2022). Drivers of adaptive evolution during chronic SARS-CoV-2 infections. Nat. Med..

[11] Borges V., Isidro J., Cunha M., Cochicho D., Martins L., Banha L., Figueiredo M., Rebelo L., Trindade M.C., Duarte S. (2021). Long-Term Evolution of SARS-CoV-2 in an Immunocompromised Patient with Non-Hodgkin Lymphoma. mSphere.

[12] Upasani V., Townsend K., Wu M.Y., Carr E.J., Hobbs A., Dowgier G., Ragno M., Herman L.S., Sharma S., Shah D. (2023). Commercial Immunoglobulin Products Contain Neutralizing Antibodies Against Severe Acute Respiratory Syndrome Coronavirus 2 Spike Protein. Clin. Infect. Dis..

[13] Turakhia Y., Thornlow B., Hinrichs A.S., De Maio N., Gozashti L., Lanfear R., Haussler D., Corbett-Detig R. (2021). Ultrafast Sample placement on Existing tRees (UShER) enables real-time phylogenetics for the SARS-CoV-2 pandemic. Nat. Genet..

[14] Elbe S., Buckland-Merrett G. (2017). Data, disease and diplomacy: GISAID’s innovative contribution to global health. Glob. Chall..

[15] Gangavarapu K., Latif A.A., Mullen J.L., Alkuzweny M., Hufbauer E., Tsueng G., Haag E., Zeller M., Aceves C.M., Zaiets K. (2023). Outbreak.info genomic reports: scalable and dynamic surveillance of SARS-CoV-2 variants and mutations. Nat. Methods.

[16] Wilkinson, S. A. J. et al. Recurrent SARS-CoV-2 Mutations in Immunodeficient Patients The COVID-19 Genomics UK (COG-UK) Consortium. https://github.com/BioWilko/recurrent-sars-cov-2-mutati. 10.1093/ve/veac050 PMC9384748 35996593

[17] Ghafari M., Hall M., Golubchik T., Ayoubkhani D., House T., MacIntyre-Cockett G., Fryer H.R., Thomson L., Nurtay A., Kemp S.A. (2024). Prevalence of persistent SARS-CoV-2 in a large community surveillance study. Nature.

[18] Korber B., Fischer W.M., Gnanakaran S., Yoon H., Theiler J., Abfalterer W., Hengartner N., Giorgi E.E., Bhattacharya T., Foley B. (2020). Tracking Changes in SARS-CoV-2 Spike: Evidence that D614G Increases Infectivity of the COVID-19 Virus. Cell.

[19] Tan C.C.S., Escalera-Zamudio M., Yavlinsky A., van Dorp L., Balloux F. (2024). Intrahost dynamics, together with genetic and phenotypic effects predict the success of viral mutations. bioRxiv.

[20] Grifoni A., Sidney J., Vita R., Peters B., Crotty S., Weiskopf D., Sette A. (2021). SARS-CoV-2 human T cell epitopes: Adaptive immune response against COVID-19. Cell Host Microbe.

[21] Bloom J.D., Neher R.A. (2023). Fitness effects of mutations to SARS-CoV-2 proteins. Virus Evol.

[22] Swadling L., Diniz M.O., Schmidt N.M., Amin O.E., Chandran A., Shaw E., Pade C., Gibbons J.M., Le Bert N., Tan A.T. (2022). Pre-Existing Polymerase-Specific T Cells Expand in Abortive Seronegative SARS-CoV-2. Nature.

[23] Tan C.C.S., Owen C.J., Tham C.Y.L., Bertoletti A., van Dorp L., Balloux F. (2021). Pre-existing T cell-mediated cross-reactivity to SARS-CoV-2 cannot solely be explained by prior exposure to endemic human coronaviruses. Infect. Genet. Evol..

[24] Tzou P.L., Tao K., Pond S.L.K., Shafer R.W. (2022). Coronavirus Resistance Database (CoV-RDB): SARS-CoV-2 susceptibility to monoclonal antibodies, convalescent plasma, and plasma from vaccinated persons. PLoS One.

[25] Nabel K.G., Clark S.A., Shankar S., Pan J., Clark L.E., Yang P., Coscia A., McKay L.G.A., Varnum H.H., Brusic V. (2022). Structural basis for continued antibody evasion by the SARS-CoV-2 receptor binding domain. Science.

[26] Baessler A., Vignali D.A.A. (2024). T Cell Exhaustion. Annu. Rev. Immunol..

[27] Tarke A., Sidney J., Kidd C.K., Dan J.M., Ramirez S.I., Yu E.D., Mateus J., da Silva Antunes R., Moore E., Rubiro P. (2021). Comprehensive analysis of T cell immunodominance and immunoprevalence of SARS-CoV-2 epitopes in COVID-19 cases. Cell Rep. Med..

[28] Reynolds C.J., Swadling L., Gibbons J.M., Pade C., Jensen M.P., Diniz M.O., Schmidt N.M., Butler D.K., Amin O.E., Bailey S.N.L. (2020). Discordant neutralizing antibody and T cell responses in asymptomatic and mild SARS-CoV-2 infection. Sci. Immunol..

[29] Wagstaffe H.R., Thwaites R.S., Sidhu J.K., Lindeboom R.G.H., Kretschmer L., Worlock K.B., Dratva L.M., Huang A., Ascough S., Papargyris L. (2025). Nat Commun. 2025. Dec 7;.

[30] Ponsford M.J., Shillitoe B.M.J., Humphreys I.R., Gennery A.R., Jolles S. (2021). COVID-19 and X-linked agammaglobulinemia (XLA) - Insights from a monogenic antibody deficiency. Curr. Opin. Allergy Clin. Immunol..

[31] Md Yusof M.Y., Arnold J., Saleem B., Vandevelde C., Dass S., Savic S., Vital E.M., Emery P. (2023). Breakthrough SARS-CoV-2 infections and prediction of moderate-to-severe outcomes during rituximab therapy in patients with rheumatic and musculoskeletal diseases in the UK: a single-centre cohort study. Lancet Rheumatol..

[32] Persistent SARS-CoV-2 infection in patients seemingly recovered from COVID-19 - Bussani - 2023 - The Journal of Pathology - Wiley Online Library. https://pathsocjournals.onlinelibrary.wiley.com/doi/full/10.1002/path.6035 10.1002/path.6035 PMC10107739 36651103

[33] Chaguza C., Hahn A.M., Petrone M.E., Zhou S., Ferguson D., Breban M.I., Pham K., Peña-Hernández M.A., Castaldi C., Hill V. (2023). Accelerated SARS-CoV-2 intrahost evolution leading to distinct genotypes during chronic infection. Cell Rep. Med..

[34] Lythgoe K.A., Hall M., Ferretti L., de Cesare M., MacIntyre-Cockett G., Trebes A., Andersson M., Otecko N., Wise E.L., Moore N. (2021). SARS-CoV-2 within-host diversity and transmission. Science.

[35] Plante J.A., Liu Y., Liu J., Xia H., Johnson B.A., Lokugamage K.G., Zhang X., Muruato A.E., Zou J., Fontes-Garfias C.R. (2021). Spike mutation D614G alters SARS-CoV-2 fitness. Nature.

[36] Kemp S.A., Collier D.A., Datir R.P., Ferreira I.A.T.M., Gayed S., Jahun A., Hosmillo M., Rees-Spear C., Mlcochova P., Lumb I.U. (2021). SARS-CoV-2 evolution during treatment of chronic infection. Nature.

[37] Chan M., Linn M.M.N., O'Hagan T., Guerra-Assunção J.A., Lackenby A., Workman S., Dacre A., Burns S.O., Breuer J., Hart J. (2023). Persistent SARS-CoV-2 PCR Positivity Despite Anti-viral Treatment in Immunodeficient Patients. J. Clin. Immunol..

[38] Shields A.M., Faustini S.E., Hill H.J., Al-Taei S., Tanner C., Ashford F., Workman S., Moreira F., Verma N., Wagg H. (2022). SARS-CoV-2 Vaccine Responses in Individuals with Antibody Deficiency: Findings from the COV-AD Study. J. Clin. Immunol..

[39] Billi B., Cholley P., Grobost V., Clément M., Rieu V., Le Guenno G., Lobbes H. (2024). Intravenous immunoglobulins for the treatment of prolonged COVID-19 in immunocompromised patients: a brief report. Front. Immunol..

[40] Gröning R., Walde J., Ahlm C., Forsell M.N.E., Normark J., Rasmuson J. (2024). Intravenous immunoglobulin therapy for COVID-19 in immunocompromised patients: A retrospective cohort study. Int. J. Infect. Dis..

[41] Hettle D., Hutchings S., Muir P., Moran E., COVID-19 Genomics UK COG-UK consortium (2022). Persistent SARS-CoV-2 infection in immunocompromised patients facilitates rapid viral evolution: Retrospective cohort study and literature review. Clin. Infect. Pract..

[42] Sixto-López Y., Correa-Basurto J., Bello M., Landeros-Rivera B., Garzón-Tiznado J.A., Montaño S. (2021). Structural insights into SARS-CoV-2 spike protein and its natural mutants found in Mexican population. Sci. Rep..

[43] Maini M.K., Pallett L.J. (2018). Defective T-cell immunity in hepatitis B virus infection: why therapeutic vaccination needs a helping hand. Lancet Gastroenterol. Hepatol..

[44] Kelly C., Swadling L., Capone S., Brown A., Richardson R., Halliday J., von Delft A., Oo Y., Mutimer D., Kurioka A. (2016). Chronic hepatitis C viral infection subverts vaccine-induced T-cell immunity in humans. Hepatology.

[45] Bosch M., Kallin N., Donakonda S., Zhang J.D., Wintersteller H., Hegenbarth S., Heim K., Ramirez C., Fürst A., Lattouf E.I. (2024). A liver immune rheostat regulates CD8 T cell immunity in chronic HBV infection. Nature.

[46] Swadling L., Halliday J., Kelly C., Brown A., Capone S., Ansari M.A., Bonsall D., Richardson R., Hartnell F., Collier J. (2016). Highly-immunogenic virally-vectored T-cell vaccines cannot overcome subversion of the T-cell response by HCV during chronic infection. Vaccines.

[47] Swadling L., Maini M.K. (2020). T cells in COVID-19 — united in diversity. Nat. Immunol..

[48] Peng Y., Mentzer A.J., Liu G., Yao X., Yin Z., Dong D., Dejnirattisai W., Rostron T., Supasa P., Liu C. (2020). Broad and strong memory CD4+ and CD8+ T cells induced by SARS-CoV-2 in UK convalescent individuals following COVID-19. Nat. Immunol..

[49] Machkovech H.M., Bedford T., Suchard M.A., Bloom J.D. (2015). Positive Selection in CD8+ T-Cell Epitopes of Influenza Virus Nucleoprotein Revealed by a Comparative Analysis of Human and Swine Viral Lineages. J. Virol..

[50] Woolthuis R.G., Van Dorp C.H., Keşmir C., De Boer R.J., Van Boven M. (2016). Long-term adaptation of the influenza A virus by escaping cytotoxic T-cell recognition. Sci. Rep..

[51] Valkenburg S.A., Quiñones-Parra S., Gras S., Komadina N., McVernon J., Wang Z., Halim H., Iannello P., Cole C., Laurie K. (2013). Acute emergence and reversion of influenza A virus quasispecies within CD8 + T cell antigenic peptides. Nat. Commun..

[52] Bowen D.G., Walker C.M. (2005). Mutational escape from CD8+ T cell immunity: HCV evolution, from chimpanzees to man. J. Exp. Med..

[53] Klenerman P., Lechner F., Kantzanou M., Ciurea A., Hengartner H., Zinkernagel R. (2000). Viral escape and the failure of cellular immune responses. Science.

[54] Rubio R., Yavlinsky A., Escalera Zamudio M., Molinos-Albert L.M., Martín Pérez C., Pradenas E., Canyelles M., Torres C., Tan C., Swadling L. (2025). Initial antigen encounter determines robust T-cell immunity against SARS-CoV-2 BA.2.86 variant three years later. J. Infect..

[55] de Silva T.I., Liu G., Lindsey B.B., Dong D., Moore S.C., Hsu N.S., Shah D., Wellington D., Mentzer A.J., Angyal A. (2021). The impact of viral mutations on recognition by SARS-CoV-2 specific T cells. iScience.

[56] Dolton G., Rius C., Hasan M.S., Wall A., Szomolay B., Behiry E., Whalley T., Southgate J., Fuller A., COVID-19 Genomics UK COG-UK consortium (2022). Emergence of immune escape at dominant SARS-CoV-2 killer T cell epitope. Cell.

[57] Le Bert N., Tan A.T., Kunasegaran K., Tham C.Y.L., Hafezi M., Chia A., Chng M.H.Y., Lin M., Tan N., Linster M. (2020). SARS-CoV-2-specific T cell immunity in cases of COVID-19 and SARS, and uninfected controls. Nature.

[58] Chen S., Zhou Y., Chen Y., Gu J. (2018). fastp: an ultra-fast all-in-one FASTQ preprocessor. Bioinformatics.

[59] Grubaugh N.D., Gangavarapu K., Quick J., Matteson N.L., De Jesus J.G., Main B.J., Tan A.L., Paul L.M., Brackney D.E., Grewal S. (2019). An amplicon-based sequencing framework for accurately measuring intrahost virus diversity using PrimalSeq and iVar. Genome Biol..

[60] O'Toole Á., Scher E., Underwood A., Jackson B., Hill V., McCrone J.T., Colquhoun R., Ruis C., Abu-Dahab K., Taylor B. (2021). Assignment of epidemiological lineages in an emerging pandemic using the pangolin tool. Virus Evol..

[61] NCBI Virus [Internet]. Bethesda (MD): National Library of Medicine (US), National Center for Biotechnology Information; 2004 – [cited 2026 01 12]. Available from: https://www.ncbi.nlm.nih.gov/labs/virus/vssi/#/

[62] Katoh K., Standley D.M. (2013). MAFFT Multiple Sequence Alignment Software Version 7: Improvements in Performance and Usability. Mol. Biol. Evol..

[63] Nguyen L.-T., Schmidt H.A., von Haeseler A., Minh B.Q. (2015). IQ-TREE: A Fast and Effective Stochastic Algorithm for Estimating Maximum-Likelihood Phylogenies. Mol. Biol. Evol..

[64] Paradis E., Schliep K. (2019). ape 5.0: an environment for modern phylogenetics and evolutionary analyses in R. Bioinformatics.

[65] Hadfield J., Megill C., Bell S.M., Huddleston J., Potter B., Callender C., Sagulenko P., Bedford T., Neher R.A. (2018). Nextstrain: real-time tracking of pathogen evolution. Bioinformatics.

[66] Vita R., Blazeska N., Marrama D., IEDB Curation Team Members, Duesing S., Bennett J., Greenbaum J., De Almeida Mendes M., Mahita J., Wheeler D.K. (2025). The Immune Epitope Database (IEDB): 2024 update. Nucleic Acids Res..

[67] Reynisson B., Alvarez B., Paul S., Peters B., Nielsen M. (2020). NetMHCpan-4.1 and NetMHCIIpan-4.0: improved predictions of MHC antigen presentation by concurrent motif deconvolution and integration of MS MHC eluted ligand data. Nucleic Acids Res..

[68] Roederer M., Nozzi J.L., Nason M.C. (2011). SPICE: Exploration and analysis of post-cytometric complex multivariate datasets. Cytometry. A..

[69] Augusto J.B., Menacho K., Andiapen M., Bowles R., Burton M., Welch S., Bhuva A.N., Seraphim A., Pade C., Joy G. (2020). Healthcare Workers Bioresource: Study outline and baseline characteristics of a prospective healthcare worker cohort to study immune protection and pathogenesis in COVID-19. Wellcome Open Res..

[70] R Core Team (2021). R: A language and environment for statistical ## computing. R Foundation for Statistical Computing, Vienna, Austria. ## URL https://www.R-project.org/

[71] Calis J.J.A., Maybeno M., Greenbaum J.A., Weiskopf D., De Silva A.D., Sette A., Keşmir C., Peters B. (2013). Properties of MHC Class I Presented Peptides That Enhance Immunogenicity. PLoS Comput. Biol..

[72] Grifoni A., Weiskopf D., Ramirez S.I., Mateus J., Dan J.M., Moderbacher C.R., Rawlings S.A., Sutherland A., Premkumar L., Jadi R.S. (2020). Targets of T Cell Responses to SARS-CoV-2 Coronavirus in Humans with COVID-19 Disease and Unexposed Individuals. Cell.

