# Supplementary material for: Extensive evolution and T cell escape by SARS-CoV-2 in a 2.5-year persistent infection of an immunocompromised host
Source: iScience. 2026 Feb 5;29(3):114917. doi: 10.1016/j.isci.2026.114917 (PMC12936837; doi:10.1016/j.isci.2026.114917)
Supplement: Document S1. Figures S1–S6 [file mmc1.pdf]

iScience, Volume *29*

Supplemental information

Extensive evolution and T cell escape by SARS-CoV-2 in a 2.5-year persistent infection of an immunocompromised host

José Afonso Guerra-Assunção, Ruairi McErlean, Katie Townsend, Selin Cankat, Leonhard M. Flaxl, Shengwei Jamie Tian, Thomas R. Turner, Neema P. Mayor, Judith Breuer, Leo Swadling, and David M. Lowe

Supplementary Figures:

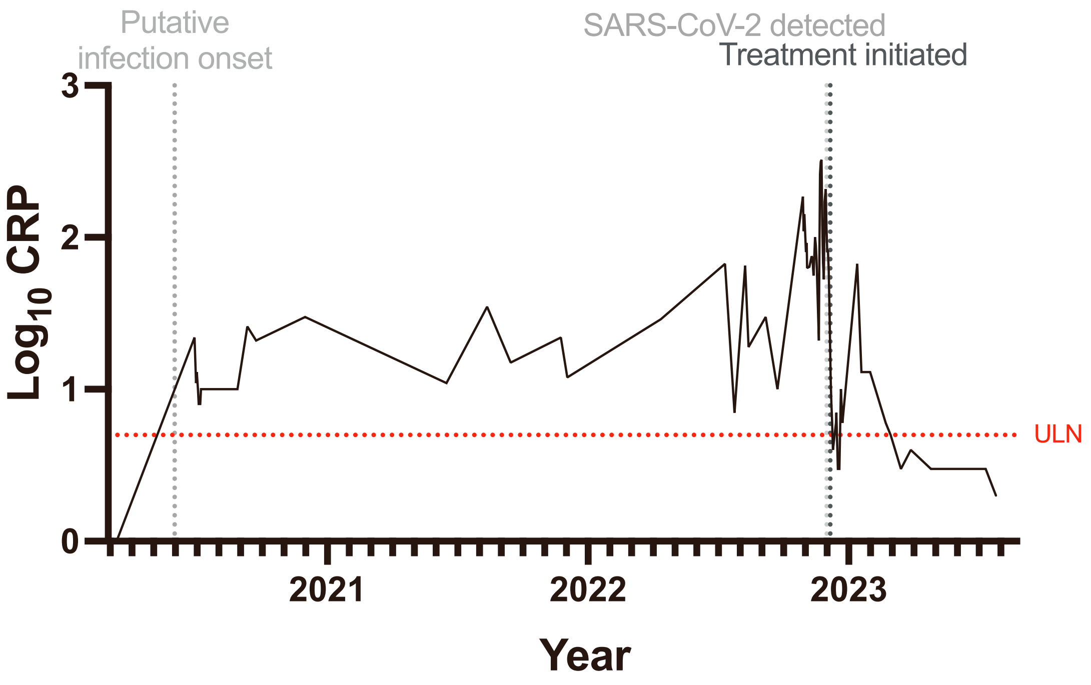

Supplementary Figure 1: Serial C-Reactive Protein measurements over infection course. The Log10 CRP values measured longitudinally with key clinical events shown with dotted vertical lines. CRP, C-reactive protein; ULN, upper limit of normality.

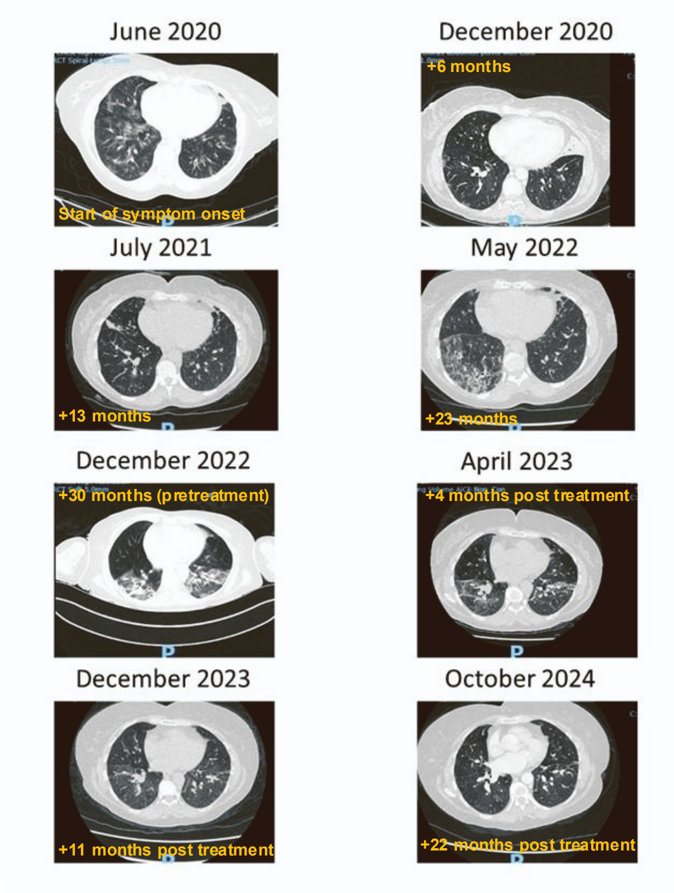

Supplementary Figure 2: Serial chest high resolution CT imaging over time. Scans from June 2020 to December 2022 demonstrate typical ground glass changes or focal areas of consolidation, consistent with COVID-19 disease. Subsequent imaging shows resolution over time but with residual fibrosis, air trapping and large airways disease.

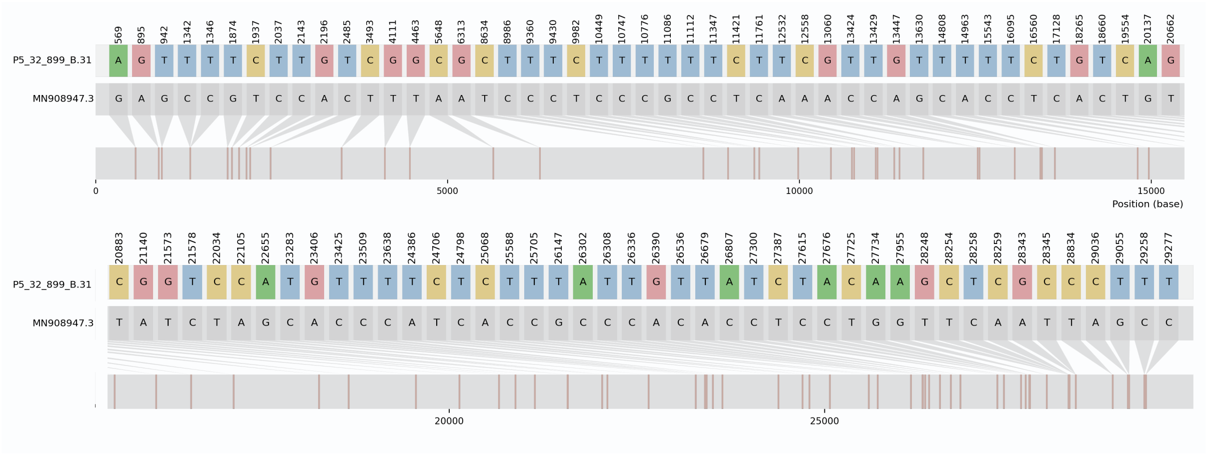

Supplementary Figure 3: Single nucleotide polymorphisms arising compared to Wuhan hu-1 reference genome (accession number, left hand side) in the virus isolated at day 899 of persistent infection.

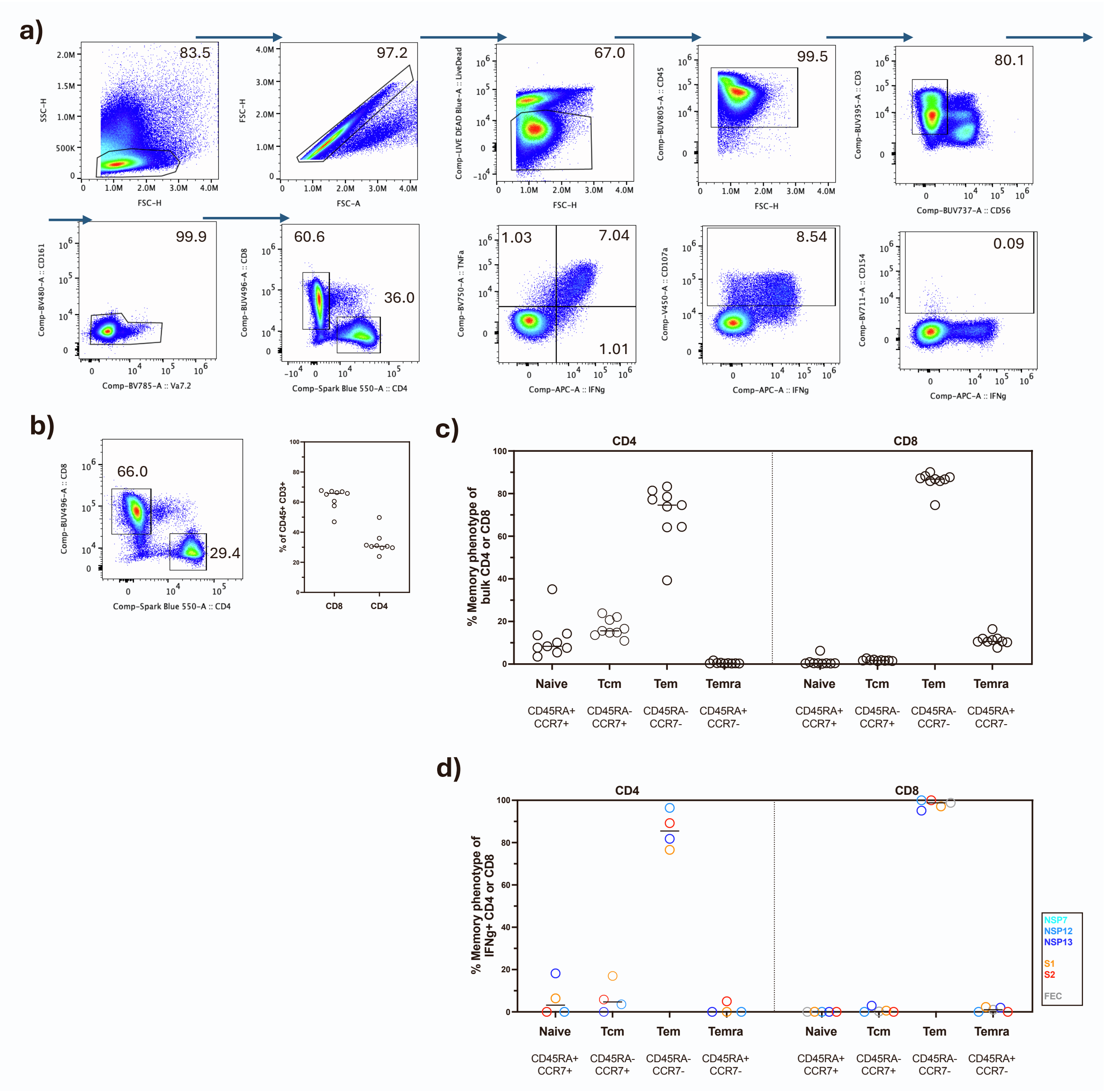

Supplementary Figure 4. Gating strategy for intracellular cytokine staining and T cell memory phenotype: a) Example FACS plots showing gating on lymphocytes (SSC-H vs. FSH-H), single cells (FSC-H vs. FSC-A), live cells (Live/dead cell permeable dye negative), CD45+, CD3+CD56- (T cells), CD161^{lo}, CD8+ or CD4+. Example plots of IFN**γ** vs. TNF, CD107a, and CD154 are shown on CD8 T cells. Stimulated with Flu, EBV, CMV epitope pool. Percentage of parent population falling in gate shown. **b)** CD4+ and CD8+ populations as a percentage of Live/CD45+/CD3+/non-MAIT T cells. **c)** Memory phenotype of bulk CD4+ and CD8+ T cells. **d)** Memory phenotype of IFN**γ**+ T cells after overnight stimulation with Spike region S1 and S2, NSP7, NSP12, NSP13 or a pool of immunodominant MHC class I-restricted Flu, EBV and CMV peptides. **c-d)** Bars, median. **b-c)** 9 replicate stains.

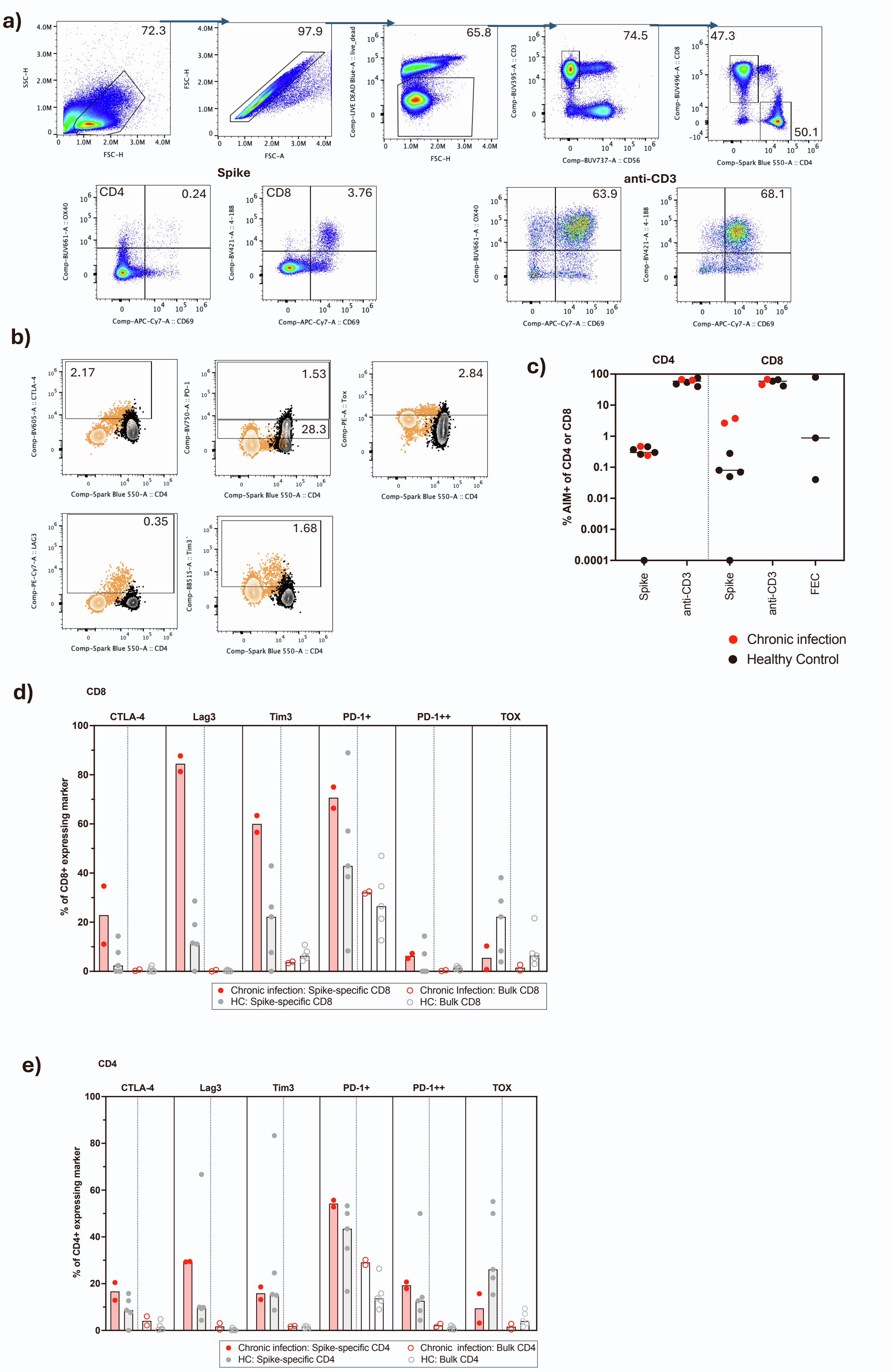

a)
72.3
97.9
65.8
74.5
47.3
50.1
SSC-H
FSC-H
FSC-A
Comp-LIVE DEAD Blue-A :: live_dead
Comp-BUV395-A :: CD3
Comp-BUV737-A :: CD56
Comp-BUV496-A :: CD8
Comp-Spark Blue 550-A :: CD4
Spike
CD4
0.24
CD8
3.76
anti-CD3
63.9
68.1
Comp-BUV661-A :: OX40
Comp-BV421-A :: 4-1BB
Comp-APC-Cy7-A :: CD69
b)
2.17
1.53
28.3
2.84
0.35
1.68
Comp-BV605-A :: CTLA-4
Comp-BV750-A :: PD-1
Comp-PE-A :: Tox
Comp-PE-Cy7-A :: LAG3
Comp-Spark Blue 550-A :: CD4
c)
CD4
CD8
% AIM+ of CD4 or CD8
Spike
anti-CD3
Spike
anti-CD3
FEC
Chronic infection
Healthy Control
d)
CD8
CTLA-4
Lag3
Tim3
PD-1+
PD-1++
TOX
% of CD8+ expressing marker
Chronic infection: Spike-specific CD8
HC: Spike-specific CD8
Chronic Infection: Bulk CD8
HC: Bulk CD8
e)
CD4
CTLA-4
Lag3
Tim3
PD-1+
PD-1++
TOX
% of CD4+ expressing marker
Chronic infection: Spike-specific CD4
HC: Spike-specific CD4
Chronic infection: Bulk CD4
HC: Bulk CD4

Supplementary Figure 5. Activation-induced marker assay: a) Example FACS plots showing gating on lymphocytes (SSC-H vs. FSH-H), single cells (FSC-H vs. FSC-A), live cells (Live/dead cell permeable dye negative), CD3+ CD56- (T cells), CD8+ or CD4+. Activated (AIM+) T cells were defined as OX40+CD69+ for CD4+ T cells and 4-1BB+CD69+ for CD8+ T cells. Example plots of PBMC from chronic infection stimulated with SARS-CoV-2 spike pool and anti-CD3 are shown below. Percentage of parent population falling in gate shown. **b)** Example plots of phenotypic and exhaustion markers in the AIM panel are overlaid for CD4 T cells (black) and CD3- (Live non-T cells; orange). Percentage of CD4 falling in positive gate shown. PD-1 expression divided into PD-1+ and PD-1++. Gates defined on Live non-CD3+ lymphocytes and CD4+ T cells. **c)** Summary data showing the magnitude of CD4 and CD8 T cell responses that expressed activation markers (AIM+; OX40+CD69+ CD4 T cells and 4-1BB+CD69+ CD8 T cells) after overnight stimulation with overlapping peptides covering SARS-CoV-2 spike (Wuhan hu-1 sequence), MHC class I restricted Flu, EBV and CMV peptides (FEC pool) or plate bound anti-CD3. The SARS-CoV-2 spike-specific memory response was measured in PBMC at two times post-infection resolution (red). Four healthy donors had previously received three spike-based vaccinations and were collected in 2025 (black). **d-e)** Bars, median. the expression of phenotypic and exhaustion markers on AIM+ and bulk CD8 **(d)** and CD4 **(e)** T cells after overnight stimulation.

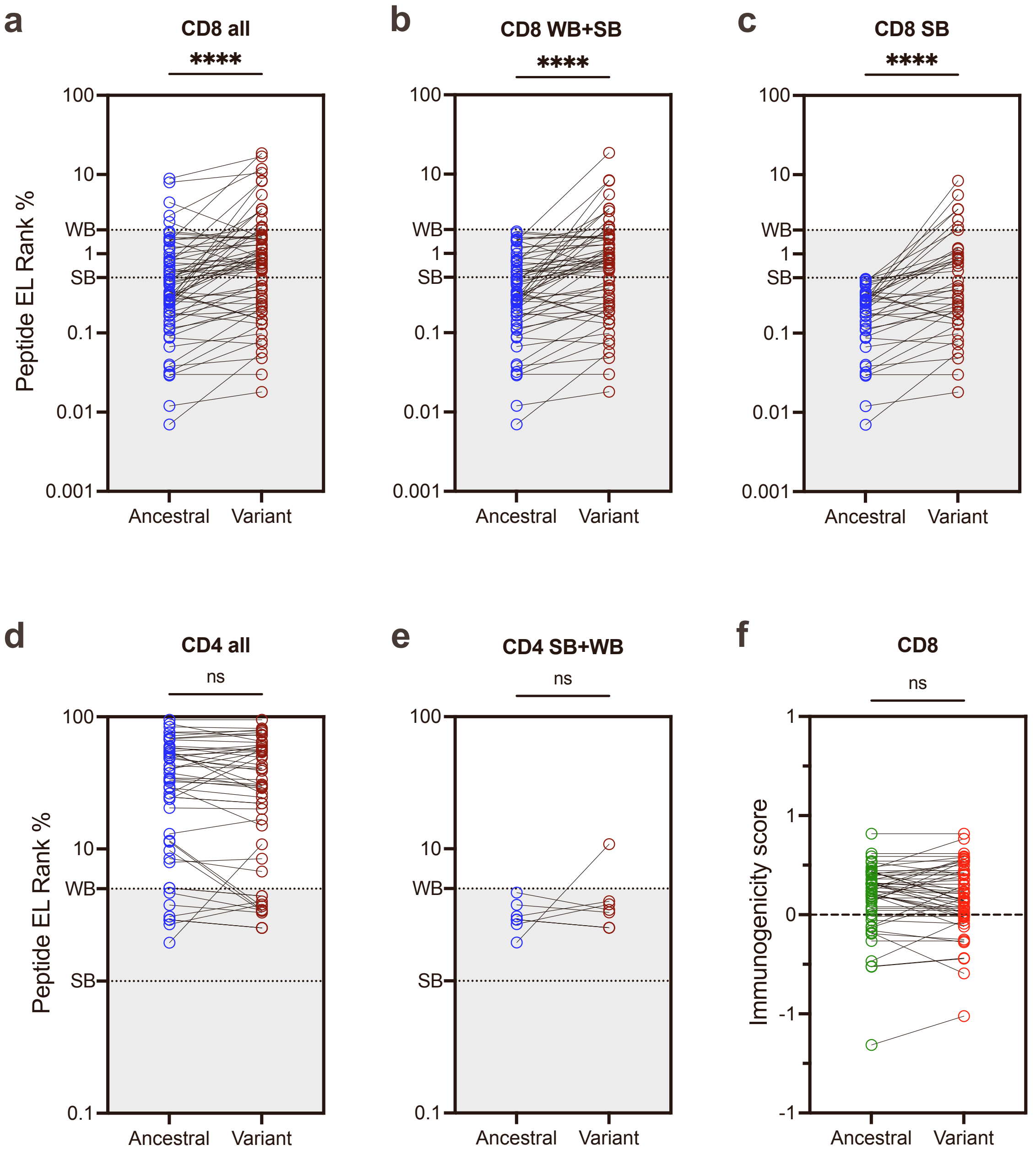

Supplementary Figure 6: Predicted impact of mutations arising during persistent infection on peptide-MHC binding and immunogenicity. a-e) The percentage elution rank (calculated using NetMHCpan or NetMHCIIpan) for validated CD8 (**a-c**; n=55) and CD4 (**d-e**; n=38) epitopes affected by non-synonymous mutations arising during this persistent infection, comparing ancestral sequence to variant sequence. **a,d**) all epitopes, (**b,e**) only those with ancestral sequence predicted as weak or strong binders (WB+SB), **c**) only strong binders. All validated HLA allele-peptide combinations are included. **f)** *In silico* predicted Immunogenicity scores of all CD8 epitopes affected by mutations arising during this persistent infection, comparing ancestral and variant sequence. Statistical analysis was performed using Wilcoxon paired non-parametric t-test. Epitope lists are given in **Supplementary Tables 3&4**.
